# Morphological diversification has led to inter-specific variation in elastic wing deformation during flight in scarab beetles

**DOI:** 10.1098/rsos.200277

**Published:** 2020-04-15

**Authors:** Y. Meresman, J. F. Husak, R. Ben-Shlomo, G. Ribak

**Affiliations:** 1 School of Zoology, Faculty of Life Sciences, Tel Aviv University, Tel Aviv 6997801, Israel; 2 Department of Biology, University of St. Thomas, Saint Paul, MN 55105, USA; 3 Department of Biology and the Environment, University of Haifa-Oranim, Tivón, Israel; 4 The Steinhardt Museum of Natural History, Israel National Center for Biodiversity Studies, Tel Aviv 6997801, Israel

**Keywords:** Coleoptera, divergent-evolution, elastic deformation, functional morphology, free flight, insect, flapping flight

## Abstract

Insect wing shapes and the internal wing-vein arrangement are remarkably diverse. Although the wings lack intrinsic musculature to adjust shape actively, they elastically deform due to aerodynamic and inertial loads during flapping. In turn, the deformations alter the shape of the wing profile affecting the aerodynamic force. To determine how changes in wing-vein arrangement affect elastic wing deformation during free flight, we compared elastic wing deformations between free-flying rose chafers (*Protaetia cuprea*) and dung beetles (*Scarabaeus puncticollis*), complementing the comparison with wing static bending measurements. The broader relevance of the results to scarab beetle divergence was examined in a geometric morphometric (GM) analysis of wing-vein arrangement in 20 species differing in phylogeny and ecology. Despite rose chafers and dung beetles demonstrating similar flapping kinematics and wing size, the rose chafer wings undergo greater elastic deformation during flapping. GM analyses corrected for phylogenetic relatedness revealed that the two beetles represent extremes in wing morphology among the scarab subfamilies. Most of the differences occur at the distal leading edge and the proximal trailing edge of the wing, diversifying the flexibility of these regions, thereby changing the pattern of elastic wing deformation during flapping. Changes to local wing compliance seem to be associated with the diversification of scarab beetles to different food sources, perhaps as an adaptation to meet the demands of diverse flight styles.

## 1. Introduction

Wing morphology has a direct effect on animal flight, and hence on the ability of flying species to exploit their environment efficiently. Trade-offs between the need to manoeuvre, hover, accelerate and fly at low energetic cost should affect wing-shape evolution and lead to diversification of wing morphology according to specific life styles. For example, high aspect-ratio (AR, wing span/wing width) wings improve energetic efficiency in gliding birds and are correlated with fast flight speeds in bats, whereas lower AR wings can enable tighter manoeuvres and low-speed flight in cluttered environments [1,2]. In insects, variation in wing morphology of true dung beetles (Scarabaeinae) suggests that varying, but unknown, selective pressures act non-uniformly on different wing regions [3], probably due to differences associated with diversification of the flight style [3–5].

Insect wings are thin structures composed of a cuticular membrane reinforced by thicker wing veins that spread span-wise and chord-wise from the wing hinge [6]. The arrangement and size of wing veins within the wing determines both the overall [7] and local (at specific wing sections) flexural rigidity and the elastic deformation of the insect wing [8–10]. During flapping, the wings are subjected to inertial and aerodynamic forces that distribute unevenly over their area. These forces elastically twist and bend the wings according to their flexural rigidity. Wing twist can compensate for the span-wise increase in the angle-of-attack (AoA) as a result of flapping, in which distal wing sections move faster than proximal sections relative to the air [11–14]. It also ensures lift production during both the upstrokes and downstrokes [13], thus distinguishing insects from flying vertebrates (reviewed by [15]). Elastic compliance of the wing also results in wing camber (curvature of the wing profile) [9,16] that may improve flow attachment to the wing, resulting in higher lift and delayed flow separation during dynamic stall and stroke reversals, where the AoA reaches ±90° [17,18]. Thus, it is generally accepted that some wing flexibility is required in flying insects. However, there is controversy as to whether wing deformations have an advantage over rigid wings in aerodynamic force production. On the one hand, studies have shown that wing flexibility can enhance load-lifting capacity [19], down-wash and lift production [20–22], delay stall during the translational phase [23,24], improve wake capture [25] and flight-efficiency [12,26], and increase tolerance to aerial perturbations, by providing more stability compared to artificially stiffer wings [27]. On the other hand, Tanaka *et al*. [28] suggested that hoverfly wings would produce greater lift if they were rigid; Zhao *et al*. [29] showed that wing flexibility reduces the generation of the aerodynamic lift; and Tobing *et al*. [30] argued that wing flexibility reduces the production of lift but enables bumblebee wings to generate thrust. This controversy may reflect trade-offs between wing flexibility and rigidity in designing efficient flapping wings but may also be partly due to research focused on simplified model wings and numerical simulations. Empirical measurements of wing deformation during flight in real insects are scarce but crucial for a true understanding of the fluid–structure interaction underlying the benefits and disadvantages of flexible wings.

The scarab family (Coleoptera: Scarabaeidae) is one of the largest and most diverse among the beetles (greater than 30 000 species). Its members differ in feeding habits (from anthophagy to coprophagy), diel activity regimes (from diurnal to nocturnal) and flight styles (steady versus highly manoeuvrable). Despite the general perception of being clumsy flyers, some scarab beetle species (e.g. flower chafers, Cetoniinae) have specialized in diurnal feeding on flowers and adopted a flight style that includes high manoeuvrability, hovering and precise landing on perches. In contrast, true dung beetles (e.g. *Scarabaeus puncticollis*) are fast, long-distance flyers that fly towards the scent of animal faeces. Upon arrival in the vicinity of the source of scent, they often cease flapping and crash-land to search for their food by walking on the ground (electronic supplementary material, film S1.1). The infra-order Scarabaeoidea appeared about 174–195 million years ago (Ma), giving rise to the Scarabaeidae and Glaphiridae. Phytophagy probably occurred first: 101–141 Ma in the Glaphiridae and 109–128 Ma in the ancestor of the Scarabaeidae subfamilies, except the Scarabaeinae. Only later did anthophagy evolve: 63–79 Ma in the Glaphiridae and 62–72 Ma in the Cetoniinae. Coprophagy probably diverged from a saprophagous ancestor and first appeared 73–82 Ma in the Scarabaeinae [31]. The homologies among the scarab beetle species nonetheless remain very clear: the wings share similar veins, enabling a one-to-one mapping of changes in wing-vein arrangement [3,32]. Since such changes can affect the wing–air interaction, they may result in a complex interplay among flexural rigidity, flapping kinematics and fluid dynamics that confine our understanding of the aerodynamic function of flexible insect wings.

During flapping, rose chafer wings undergo massive chord-wise deformation (camber) with a clear span-wise gradient (wing twist) [9]. Here, we examine whether these large wing deformations are due to a morphological wing change that might have emerged during the diversification of flower chafers from other scarab beetles. Modification of insect wing shape, on an evolutionary scale, could occur via a rearrangement of wing veins, thus altering both geometrical wing properties (planform shape, area, AR) as well as the structural properties affecting rigidity [19]. Bai *et al*. [33] found that true dung beetle wings demonstrate a higher morphological variance in regions proximal to the wing base, whereas the distal part of the wing is conserved. However, the relationship between inter-specific differences in local wing-vein arrangement and elastic wing deformation during flapping is poorly understood.

Shahzad *et al*. [34] showed that while flexible flapping wings with higher AR and lower distribution of wing area towards the wing base tend to generate larger lift forces during hovering, they typically do not outperform rigid wings. In contrast, flexible wings with lower AR and more of the wing area distributed towards the wing base tend to have higher power efficiency during hovering. Wing compliance was also found to be beneficial for power saving during slow flight and hovering, but required higher power (compared to a rigid wing) during fast forward flight [35]. Other studies have argued that the higher camber at mid-stroke and steeper wing twist at stroke reversals could be more efficient for hovering or flying at slow speeds [15,25]. Together, these studies suggest that those insects better adapted for hovering and low-speed flight (e.g. flower chafers) would benefit more from having compliant wings; while insects better adapted for straight flight at higher speed (e.g. dung beetles) would benefit from their wings being more rigid.

Based on this idea, we hypothesized that the diversification of scarab beetles was associated with changes to the mechanical properties of their wings via wing-vein arrangement leading to flower chafer wings being more flexible than dung beetle wings. To test this hypothesis, we compared the actual wing deformation during free flight between *Protaetia cuprea* (Cetoniinae) and *S. puncticollis* (Scarabaeinae) as representatives of two extreme wing shapes within the scarab beetle family. We then performed static bending measurements on the wings of the two species and supplemented the analysis with a geometric morphometric (GM) study of variance in wing-vein arrangement in 20 diverged scarab beetle species.

## 2. Material and methods

### 2.1. Insects

We collected 21 adult dung beetles (*S. puncticollis*; 14 female, 6 male, 1 unidentified sex) from the Nitzanim Nature Reserve (31°45' N, 34°37' E). Beetle body mass ranged between 0.198 and 0.908 g and wing length ranged between 14 and 25 mm, in the smallest and largest beetle, respectively. Inter-sex differences in body mass and wing length were insignificant (two-tailed *t*-test; *t*_18_ = 0.772, *p* = 0.450 and *t*_18_ = 1.339, *p* = 0.197, respectively); therefore, data from both sexes were pooled. The free-flight rose chafer (*P. cuprea*) data were taken from a previous study [9] employing the same research set-up, while the rose chafers for the static wing bending measurements (see below) came from a lab population reared at Tel-Aviv University.

### 2.2. Measuring wing deformation during free flight

**Figure 1.**
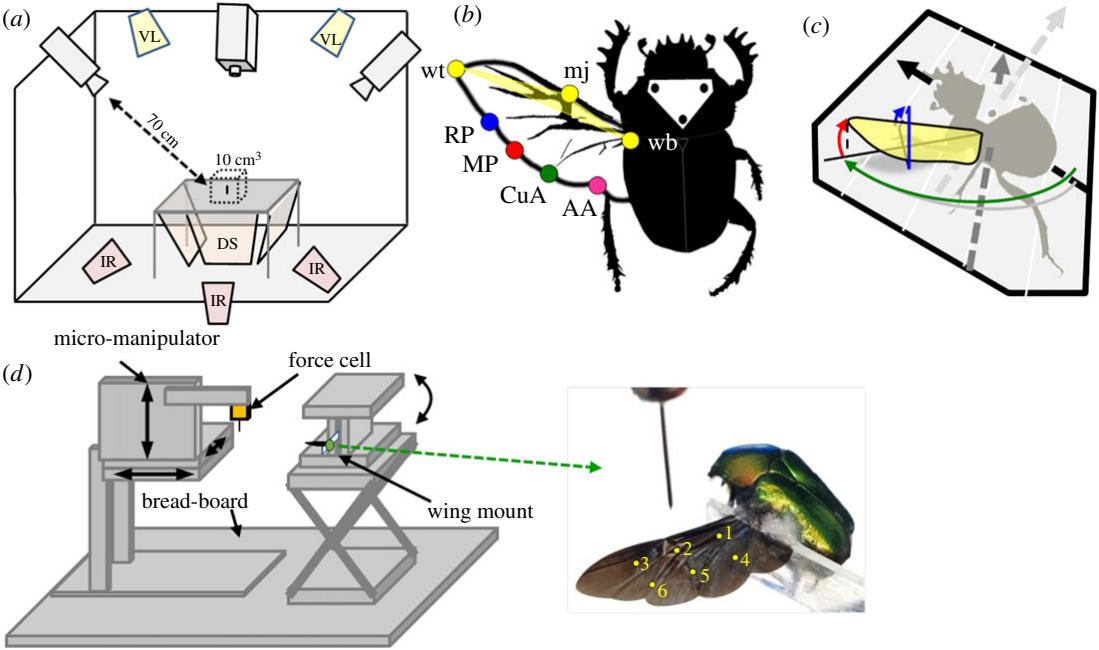
Methodology illustrations. (*a*) The flight arena. VL: visual light; IR: infrared light; DS: diffusive screen. (*b*) The dung beetle *S. puncticollis* with the left wing extended. Shown are three dots marked on the pronotum and seven landmarks on the wing denoted by letters. (*c*) Definition of the three instantaneous angles used to depict flapping kinematics: incidence (blue), flapping (green) and deviation (red). Dashed arrows denote the body axes. (*d*) A schematic illustration (not to scale) of the force measurement set-up. Beetles were secured to a custom-built mount that enabled inverting the beetle while still attached. The wings were pressed down at six points (yellow dots on the right image).

Eighteen free-flying dung beetles were filmed using three high-speed cameras. For each individual, a single film was chosen showing the beetle flying at low speed well above the ground and performing at least three symmetrical flapping cycles (electronic supplementary material, film S1.2). The filming set-up procedure and subsequent analyses to extract flight speed and acceleration, flapping kinematics (as three time-varying angles) and wing deformation (figure 1*a–c*), were identical to those in [9], allowing us to compare the flapping kinematics and wing deformations between species. Briefly, we used three points on the thorax to extract the body orientation in the films and seven landmarks on the left wing to extract the wing flapping kinematics relative to the body and the elastic wing deformations. Three of the wing landmarks were on the leading edge (wb, mj and wt in figure 1*b*). They were used to define a rigid wing plane in each movie frame. Next, we measured the local chord-wise wing compliance as the deflection of four trailing edge landmarks (RP, MP, CuA and AA in figure 1*b*) relative (perpendicular) to the leading edge plane (electronic supplementary material, S2.1). Various sources of measurement error are either evaluated and accounted for or shown to be negligible in electronic supplementary material, S2.2.

### 2.3. Static bending measurements

The local flexural stiffness of the wing was measured in five rose chafers and six dung beetles. We secured live beetles at their wing base to a custom-built apparatus, with one wing stretched in the air as a cantilever beam. We used a force transducer, fitted with a pin, to press the wings down 1 mm, thus measuring both displacement and the resulting force. The procedure was repeated at six locations on the wing (figure 1*d*; electronic supplementary material, S2.3). Subsequently, we added an additional support under the entire leading edge, restricting span-wise bending and wing twist. Finally, the mounted beetle was inverted (upside-down) to enable application of force on the same locations from the ventral side of the wing. Hence, each wing was tested four times: pressing from the dorsal (1) and ventral (2) sides, and while the wing was secured as a cantilever beam only by the wing base (hereafter ‘WB1’ and ‘WB2’); and again, with the additional leading edge (LE) support (hereafter ‘WBLE1’ and ‘WBLE2’). We repeated the WB1 and WBLE1 measurements on another eight *P. cuprea* in order to increase the distribution of points where force was applied. The flexural stiffness (*EI*) of a uniform cantilever beam can be calculated from the relationship between the deflection (*β*) caused by applying a point load (force, *F*) at a specific distance (*l*) from a fixed support [36], as

2.1 $$\beta =\frac{Fl^{3}}{kEI}$$

where *k* is a constant. Since in both wings the deflection (1 mm) and distance between the support and the point of force application (*l*) were the same, the measured force required to bend the wing is proportional to the flexural stiffness of the wing, for each point.

### 2.4. Beetle collection and handling for wing morphometry

**Figure 2.**
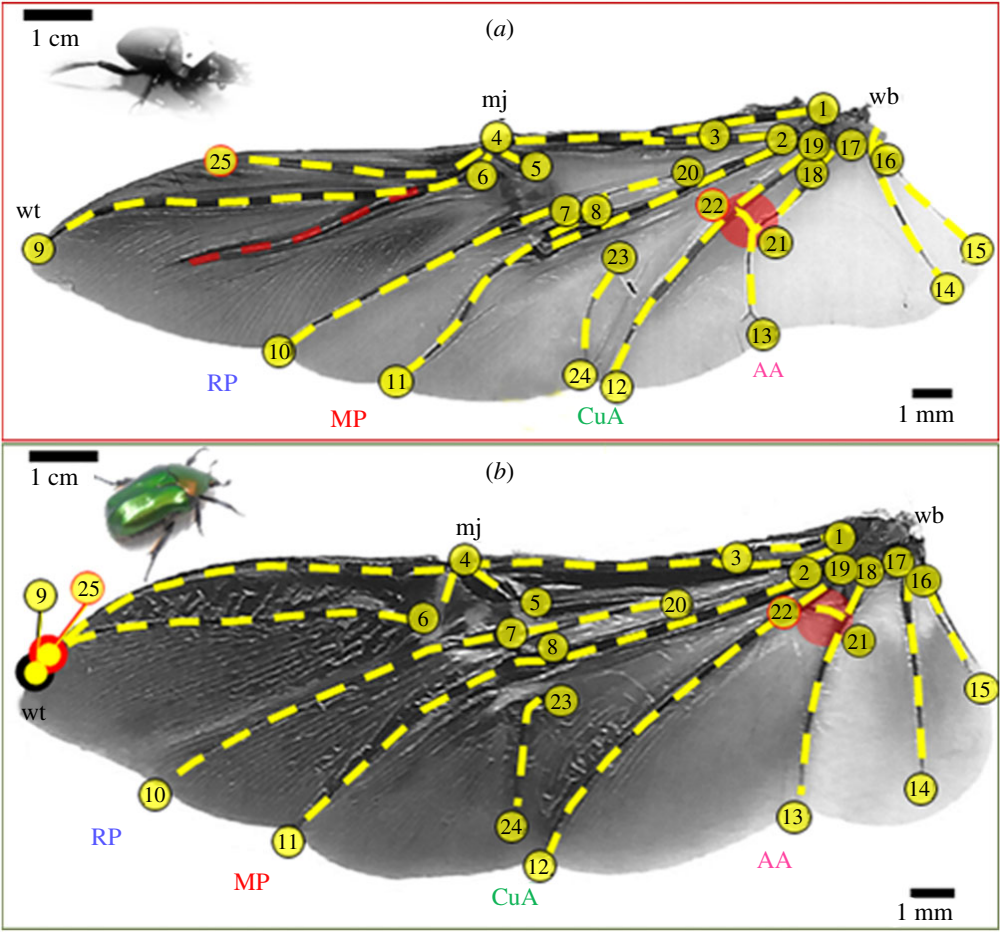
The wings of the (*a*) dung beetle and (*b*) rose chafer. Numbered circles denote the 25 homologous landmarks used in the GM analyses. Wing veins are highlighted in yellow dashed lines. Specific veins discussed in the text are marked in red.

For the morphological study 203 beetles from 20 species belonging to five subfamilies of the Scarabaeidae were collected at various sites in Israel (forests, sand dunes and arid regions). For each species we noted the adult feeding preferences, diel activity and the posture of the elytra during flight (electronic supplementary material, S3). The beetles were euthanized in ethyl-acetate and their left wing was removed, stretched over a transparent sheet and scanned (HP Officejet 6700 premium) to a resolution of 1200 or 2400 dpi, depending on wing size. Using ImageJ (v. 1.51 K) we digitized 25 clearly distinguishable homologous landmarks on the wing-vein structure of each image (figure 2). We performed a Procrustes analysis (reviewed by Adams *et al*. [37]) based on the two-dimensional coordinates of the landmarks followed by a principal components analysis (PCA) using the ‘procGPA’ function in the ‘Shapes’ package in R [38]. Next, to separate inter-specific variance in wing shape from phylogenetic relatedness, we performed a phylogeny-corrected PCA based on the same landmarks using the ‘Phylo.pca’ function in ‘Phytools’ R package [39], and the phylogenetic data on the 20 species detailed in electronic supplementary material, S4. For *S. puncticollis* and *P. cuprea*, we measured wing span (*b*) and area (*S*) directly from the scaled wing images using ImageJ and calculated the AR as AR = *b*^2^/*S*. The second moment of wing area was calculated with a custom-written Matlab (The Mathworks Inc.) code as in [40].

### 2.5. Statistical analysis

Repeated-measures ANOVA (RMANOVA) was performed to determine whether the different trailing edge landmarks deflected with different magnitudes relative to each other during the downstroke in *S. puncticollis*. Because sphericity (Mauchly's test) could not be assumed, we used the Greenhouse–Geisser correction to interpret the results. As the upstroke deflections were not normally distributed, we performed the Friedman ANOVA as an alternative to RMANOVA. *Post hoc* comparisons of specific wing landmark deflections in *S. puncticollis* were performed using paired-samples *t*-test and Wilcoxon *z*-test for downstroke and upstroke deflections, respectively. To compare the deflections of specific wing landmarks between free-flying rose chafers and dung beetles, we used either independent-samples two-tailed *t*-tests or Mann–Whitney *U*-tests according to test assumptions. Significance levels were Bonferroni corrected to account for multiple comparisons.

In the static bending tests, for each species, we used paired-samples *t*-tests to compare the force-specific deflection between dorsal and ventral treatments and WB versus WBLE treatments at each point separately. To compare the force-specific deflections in the WB experiments between the species we used independent-samples *t*-tests.

To compare the mean wing shape of the rose chafer, *P. cuprea*, against that of the true dung beetle, *S. puncticollis*, we applied thin plate spline (TPS) analysis using the ‘Shapes’ package in R [38]. TPS analysis enables visualization of the trajectories between homologous landmarks of the mean wing shape following Procrustes correction. Although comparing two species limits the ability to elucidate evolutionary patterns [41], these species are representatives of their respective subfamilies, and we supplemented the comparison between the two species with the larger context of 18 other species.

## 3. Results

### 3.1. Wing morphology and flapping kinematics in *S. puncticollis* and *P. cuprea*

**Figure 3.**
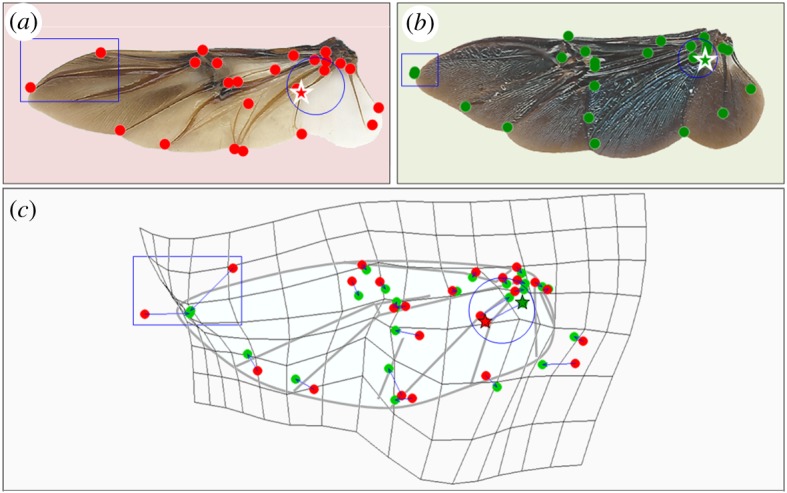
(*a*–*c*) Thin plate spline. A comparison of the homologous landmarks distribution between wings of dung beetles (red) and of rose chafers (green). Blue arrows show trajectories of the landmarks from dung beetle and rose chafer wings. The grid shows areas of contraction and expansion among the landmarks. The circle and rectangle show areas with the most variance in the phylo-PCA (i.e. highest loadings). Landmark 22 is denoted by a star.

Both *P. cuprea* and *S. puncticollis* displayed similar body mass and wing size. The relative distribution of wing mass along the span-wise and chord-wise axes was also comparable (electronic supplementary material, S5). However, the dung beetles had higher AR wings with more of the wing area distributed distally compared with the rose chafer wings (figure 2; electronic supplementary material, S6). The TPS analysis comparing rose chafer and dung beetle wing-vein arrangements revealed that, in the former, two landmarks at the distal section of the leading edge converged (9 and 25 in figure 2; rectangle in figure 3), whereas at the proximal section of the wing, landmarks 12 and 21–24 shifted apart from one another (circle in figure 3).

The flapping kinematics and flight speeds of both species were remarkably similar (electronic supplementary material, S6), despite *S. puncticollis* revealing a slightly higher average stroke plane angle, (34° ± 1.5 versus 30° ± 0.7, *p* = 0.008), wingbeat frequency (118 Hz ± 1.8 versus 109 Hz ± 2.3, *p* < 0.001) and vertical speed (0.41 m s^−1^ ± 0.03 versus 0.29 m s^−1^ ± 0.03, *p* = 0.009). Both species had advance ratios (flight speed/wing flapping speed) much lower than 0.1 (electronic supplementary material, S6) enabling us to treat the flights as hovering conditions [42].

### 3.2. Wing deformation

**Figure 4.**
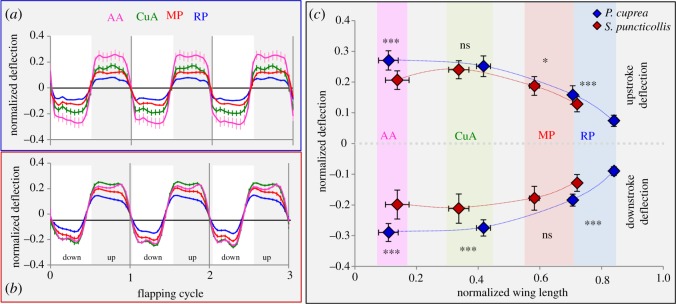
Wing deformation during flapping. Mean deflections (error bars are ±1 s.e.) of the trailing edge during three flapping cycles of free-flying (*a*) rose chafers and (*b*) dung beetles. (*c*) Mean deflections in mid-downstrokes and -upstrokes of both species according to the distribution of the trailing edge wing-vein tips RP, MP, CuA and AA (figure 1*b* for landmark positions). The colour-coded lines drawn among the landmarks are interpolations to visualize span-wise gradient in chord-wise deflection. Asterisks denote significance of interspecies difference in *t*-tests (downstroke) and Mann–Whitney *U*-test (upstroke), see electronic supplementary material, S7.2 for additional data.

Throughout the flapping cycle, the deformation of the dung beetle wing demonstrated a cyclical pattern similar to that of the rose chafer (figure 4*a,b*), with the four trailing edge (TE) landmarks (RP, MP, CuA and AA, figure 1*b*) deflecting out of plane towards the direction of the wing's movement (i.e. towards the pressure side of the wing). The magnitude of the deflections (normalized by wing length) at mid-stroke significantly differed among the TE landmarks, during both downstroke (RMANOVA, d.f. = 1.402, *F* = 176.469, *p* < 0.001) and upstroke (Friedman test, d.f. = 3, *χ*^2^ = 51.126, *p* < 0.001), with the deflection of landmark CuA being the highest (figure 4*b,c*; electronic supplementary material, S7). Deflection of the same TE landmarks significantly differed between the two species, with rose chafer wings revealing a larger deflection of the proximal trailing edge (figure 4; electronic supplementary material, S7.2).

To correct for variance in body size and vertical acceleration, we examined the differences in the allometry of trailing-edge deformation between the two species, using the vertical force needed to maintain the observed flight [Fv = body mass × (gravity + vertical acceleration)] as the predictor. Only the deflection at the proximal landmark (AA) was linearly related to Fv (after log transformation) in both species; and the homogeneity of variance assumption was rejected (Levene's test, *F*_1,32_ = 5.4, *p* = 0.027), preventing analysis of covariance. Nonetheless, we noted that the deflection (*β*) of the proximal part of the wing increased with increasing *F_v_* , according to $\beta =0.038F_{v}^{0.43\;(0.001\;\text{–}\;0.859)\;}$ in dung beetles and $\beta =0.034F_{v}^{0.34\;(0.134\;\text{–}\;0.545)\;}$ in rose chafers, with a large overlap of the 95% confidence intervals (shown in the parentheses above) of the two slopes. Hence, while deflection increases with body mass and vertical acceleration similarly in the two species, the wing deformations of rose chafers are larger on average (electronic supplementary material, S7.3).

### 3.3. Static bending measurements

**Figure 5.**
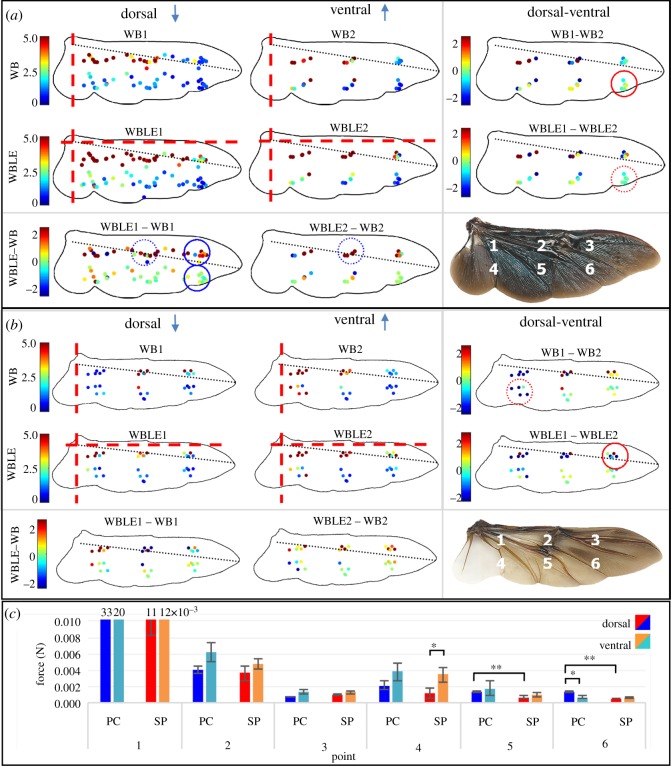
Static bending. Force measurements (colour table, in N) from wings of (*a*) *P. cuprea* and (*b*) *S. puncticollis*. WB and WBLE correspond to supporting the wing at its base or at both the wing base and leading edge, respectively, as described in the text. Numbers correspond to measuring while applying force on the dorsal (1) or ventral (2) sides of the wing. Right-hand panels show differences between dorsal and (minus) ventral sides in the WB and WBLE experiments. Bottom panels show differences between the two wing supports, WBLE minus WB in the ventral and dorsal experiments. Red and blue circles denote statistical significance in the ‘sides’ and ‘support’ comparisons, respectively. (*c*) A summary of the comparisons (*t*-test) at each point both within and between species in the WB experiments. *p < 0.05, **p < 0.01. See electronic supplementary material, S2.3 for statistical data.

Both species’ wings demonstrated a gradient in wing stiffness that decreased from wing base towards wing tip and from the leading edge to the trailing edge (figure 5). The estimation of the mean (±s.e.) chord-wise flexural stiffness (*EI*) while applying a point load at 0.42 of the wing length and 0.5 (*P. cuprea*) and 0.57 (*S. puncticollis*) of the chord length (point 5 in figure 1*d*) was estimated at 3 × 10^−8^ N m^2^ (±3 × 10^−9^) and 1 × 10^−8^ N m^2^ (±4 × 10^−9^) in *P. cuprea* and *S. puncticollis*, respectively, in the WBLE1 experiment. These estimates based on equation (2.1) and assuming *k* = 3 for a uniform cantilever beam are an order of magnitude lower than predicted based on scaling derived from insects other than beetles [7].

The force required to bend the rose chafer wing when pressing at points 2 and 3 was significantly higher using the WBLE support compared with the WB support (paired *t*-tests, *p* < 0.05; see electronic supplementary material, S2.3.3 for detailed statistics). In all other points of the rose chafer wing and all points on the dung beetle wing, the type of support did not significantly affect the magnitude of force needed to bend the wings (figure 5).

In the WBLE experiment, the force required to bend the wings at points 1–2 (proximal leading edge) was generally higher in the rose chafer, compared with the dung beetle, but the difference diminished closer to the wing tip (point 3) and in points 4–6 that were closer to the trailing edge (figure 5, electronic supplementary material, S7.4).

In the WB experiment, differences in flexural stiffness between the beetles were statistically significant only at points 4–6, closer to the trailing edge, with the rose chafer wing requiring higher forces to bend at points 5 and 6 compared with the dung beetle wing, but only when pressed from the dorsal side (figure 5*c*, electronic supplementary material, S2). In the proximal wing part (point 4), the forces required to bend the wing of the two beetles did not vary. However, at point 4 the force did not vary between pressing on the dorsal and ventral sides in the rose chafer wing, whereas in the dung beetle the force was significantly higher when applied at the ventral side compared with the dorsal side (electronic supplementary material, S2.3). At distal point 6, the pattern was the opposite, i.e. the stiffness of the rose chafer wing was dorso-ventrally asymmetric, while in the dung beetle it was not (figure 5, electronic supplementary material, S2.3).

### 3.4. Wing morphology in the scarab family

**Figure 6.**
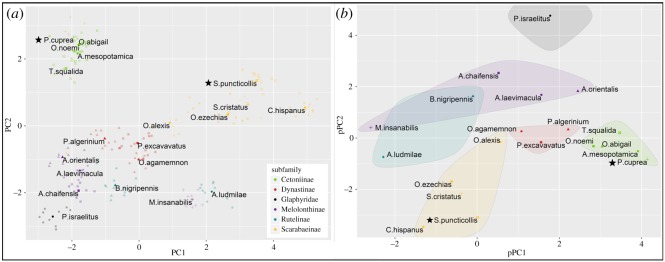
Geometric-morphometrics of scarab wings. (*a*) Principle component analysis. Each semitransparent point represents a single wing. Members of the same subfamily share the same colour. Members of the same species share both colour and symbol. The mean point for each species is marked with an opaque colour and accompanied with the species' name. Stars mark the rose chafer, *P. cuprea*, and the dung beetle, *S. puncticollis*. (*b*) Phylogenetically corrected PCA. Only the mean of each species is presented. Shapes and colours are as in (*a*). All members of the same subfamily are encircled together.

The PCA based on the wings of the 20 scarab species showed larger variance among subfamilies than within them (figure 6*a*), with a few exceptions (below). The first two principle component axes explained 49.6% (PC1) and 21.8% (PC2) of the variance in hind-wing venal arrangement. After correcting the PCA analysis for phylogenetic relatedness based on molecular data (electronic supplementary material, S4), we found a similar diversification of wing shape according to subfamily (figure 6*b*). However, while the wings of the Cetoniinae, Dynastinae and Scarabaeine subfamilies were grouped apart from each other, the wings of the Melolonthinae and Rutelinae were indistinguishable based on the first two PC axes. Species from these two latter subfamilies appeared to be ‘taxonomically misplaced’ also in the phylogenetic analysis based on molecular data (electronic supplementary material, S4.4). The outgroup, *Pygopleurus israelitus* (Coleoptera: Glaphyridae), differed from the Scarabaeidae group both before and after phylogenetic correction (figure 6*a,b*).

The distribution of the phylo-PC1 loadings revealed that landmarks 22 and 25 (red circles in figure 2) contributed the most to the difference among wings, explaining 8.9% and 9.2%, respectively, of the total variance in the 25 landmarks (electronic supplementary material, S7.5). These landmarks are located on the distal leading edge and on the vein that reinforces the proximal trailing edge of the wing (landmarks 25 and 22, respectively).

## 4. Discussion

Wing compliance is a dominant property in insect flapping flight. Our study demonstrates that morphological wing diversification can vary beyond that of the obvious changes in wing planform shape, relative size and flapping kinematics to include subtle shifts in specific wing-vein arrangement. In turn, these shifts affect local wing rigidity, thus dynamically changing the wing profile during flapping flight differently among species. Indeed, we found that morphological wing change is associated with the wings of free-flying rose chafers undergoing greater deformations at the trailing edge compared with dung beetle wings, despite having a more rigid leading edge (elaborated on below). Previous studies have shown that with appropriate flapping kinematics, wing compliance can improve lift, alter the lift-to-drag ratio and reduce power input per amount of lift generated compared with rigid wings [12,34,43]. Therefore, subtle changes in local wing compliance should be naturally selected as part of species diversification.

### 4.1. Wing morphology

**Figure 7.**
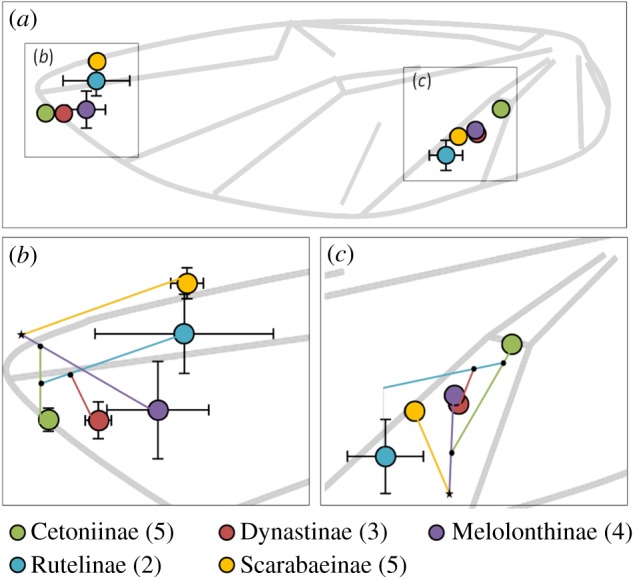
(*a*) Normalized location (±s.e.) of landmarks 22 and 25 demonstrating divergence of wing-vein arrangement in the different subfamilies. The two landmarks showed the highest PC scores in the GM analysis of scarab beetle wings. They are shown superimposed on a schematic illustration of a wing. The sample size (species) for each subfamily is shown in parentheses. The lower panel illustrates magnified areas around landmark 25 (*b*) and 22 (*c*). Coloured lines represent phylogenetic relatedness according to the phylogenetic tree in electronic supplementary material, S4.1.4. Black dots represent tree nodes but do not imply the ancestral form. The star denotes the ancestral node of all the subfamilies.

On an evolutionary scale, shifts in the venal arrangement can be governed by regulatory genes during development and affect flight performance [44]. These changes can alter the wing's elastic properties and the overall aerofoil shape during flight [13]. In the broader context of 20 beetle species, most of the represented subfamilies were distinguishable in the GM analysis based on their wing-vein arrangement (but see electronic supplementary material, S4.4). The inter-vein junction between the cubitus-anterior and the analis-anterior veins (landmark 22, red circle in figure 2) had undergone modification during the diversification of the scarab beetles (figure 7). The shift of this reinforcement towards the wing base in the Cetoniinae (flower chafers) has resulted in a more compliant region.

In contrast, the Scarabaeinae (true dung beetles) wings have an additional vein that diverges from the radius–anterior vein (red, dashed line in figure 2*a*; RA4+RP1 vein, see [32]). This vein, absent in other subfamilies, probably provides additional support to the distal wing section against span- and chord-wise bending. Its added rigidity might have enabled the shifting of the adjacent landmarks (25 and 10) away from the wing tip (figures 2 and 7), resulting in the narrow and higher AR wings of true dung beetles, distinguishing them from other subfamilies.

The divergence of scarab beetles is associated with speciation to different food resources [31]. Targeting different resources might have led the way to diversification of wing morphology. However, other differences in life habits may also impose selection forces on wing shape, thus masking the morphological divergence due to feeding preference. For instance, nocturnal activity demands adjustments for flight under different visibility conditions [4]. Low light intensity can lead to reduced flight speed in order to improve image sharpness, despite the prolonged light integration time in the photoreceptors [45–47]. The posture of the elytra during flight also varies among species and should affect flight performance. Closed elytra during flight are associated, in scarab beetles, with targeting ephemeral resources such as dung and flowers [48]. Such variation in diel activity and elytra position within the subfamilies (electronic supplementary material, S3) is circumvented in our study by focusing on two beetle species (*P. cuprea* and *S. puncticollis*) that share similarity in body size, flapping kinematics, diurnal activity and elytra opening, despite having their ancestors diverged about 160 Ma to different lineages specializing on targeting different food resources [31]. The two species, thus, represent phylogenetic differences in wing shape and compliance that are unrelated to elytra position and/or diel activity.

The variation in the proximal wing reinforcement appears to have an important role in the wing compliance difference between dung beetles and flower chafers, allowing the rose chafer (but not the dung beetle) wings to flex evenly to both dorsal and ventral sides (figure 5, point 4). Moreover, the occurrence of the additional vein in the true dung beetles is accompanied by small shifts in the trailing edge veins towards the wing base. Together with the proximal inter-vein junction (landmarks 21–22) moving towards the trailing edge in *S. puncticollis*, these anatomical adjustments alter the distribution of the dung beetle wing stiffening compared with that in rose chafers, yielding smaller trailing edge deformations during flapping. It is important to note that the observed interspecies differences in deflection at the CuA and MP landmarks during free flight are under-estimated due to the deflection increasing towards the wing base in both beetles and the same landmarks being closer to the wing base in dung beetles (figure 4*c*).

### 4.2. Functional morphology

During flight, the wings of both species elastically deform. However, despite having similar wing loading and flapping kinematics, rose chafer wings undergo larger elastic deformations compared with dung beetle wings. The chord length at the proximal part of the wings is longer in the rose chafer (figure 3, electronic supplementary material, S7.6), contributing to larger deflection in this region (figure 4) and to lowering the AR and moment of wing area of the entire wing. To evaluate if the longer chords are the sole contributor to the larger trailing edge deflections in the rose chafer we corrected for chord length in the cantilever beam assumption as follows: we note from equation (2.1) that if the force responsible for bending is equal, the flexural stiffness (*EI*) of the local wing chord is proportional to the cubic-power of the local chord length (*l*) and the measured deflection of the landmark (*β*) [7], i.e.

$$EI\propto \frac{l^{3}}{\beta }.$$

The assumption of similar force is based on the similar wing loading and wing area in the two species. In the proximal wing landmarks of the dung beetle, the ratio of $l^{3}/\beta$ is significantly larger than that of the rose chafer during the upstroke but not the downstroke (electronic supplementary material, S7.7). This indicates that unless the local forces are higher in the dung beetle, both reduced flexural stiffness and longer chords contribute to the larger deformation of the proximal trailing edge in the rose chafer. This larger deformation (empirically as well as in proportion to the chord length, electronic supplementary material, S7.8), results in larger twist and camber in the proximal rose chafer wing. Thus, the larger wing deformation supports our hypothesis of flower chafers having more compliant wings that are suited for low speed and hovering flight.

The static bending tests contribute to the analysis of wing compliance by measuring the *EI* of wing sections closer to the leading edge. It showed that the proximal leading edge of the rose chafer is stiffer than that of the dung beetle but the difference in stiffness quickly diminishes at about half the chord length (electronic supplementary material, S7.4). The steeper chord-wise gradient in the flexural stiffness of the rose chafer seems to continue beyond the half chord, leading to the observed larger trailing edge deflection and the lower flexural stiffness calculated from it, in the free-flight deformations. Interestingly, in the rose chafer wing, forces required to bend the wing close to the leading edge (points 2 and 3) under static load were higher when the wing was secured simultaneously by the leading edge and the wing base (WBLE) while in the dung beetles adding support under the leading edge did not increase the flexural stiffness of the wing beyond that of the WB measurement. Securing the leading edge primarily limits the wing's ability to twist when pressed upon, suggesting that the rose chafer wing may be more prone to twisting under load.

The higher measurement of flexural stiffness in the rose chafer leading edge is also contributed by the fact that the measurements 2 and 4 mm from the leading edge (points 1–3 and 4–6 in figure 1*d*, respectively) represent a proportionally smaller chord-wise distance in the rose chafer wing which is of lower AR. In other words, for a beetle with a wing length of 2 cm, points 4–6, 4 mm away from the leading edge, are on average 14.5% closer to the rigid leading edge in the rose chafer compared with the dung beetle, contributing to the appearance of higher rigidity in the rose chafer wing.

The static bending measurements also revealed stronger anisotropy in the proximal section of the dung beetle wing. The wing was stiffer when bending it from the ventral side, while in the proximal section of the rose chafer wing the bending ventrally and dorsally were symmetrical (figure 5). Dorso-ventral asymmetry in trailing edge deformation has also been described in other insects [13,49,50]. Such a mechanical property could be advantageous for forward flight, since during fast forward flight the inflow induces force asymmetry between the downstrokes and upstrokes [51]. However, during low-speed flight or hovering with a horizontal stroke plane, the inflow has negligible effect, favouring symmetry in wing deformation between the upstroke and downstroke. Walker *et al*. [52] hypothesized that a hinged flap at the base of hoverfly wing (i.e. the alula) may act as a flow-control device. Perhaps, the flexible proximal wing section of the rose chafer has a similar function to aid in flights requiring precise control for landing and flight stability during hovering. Dorso-ventrally symmetric wing compliance can also improve flight stability at low speed by attenuating perturbations acting on a single wing. In bumblebees, wing flexibility improves flight stability in wind [27], which may be more important for insects needing to perform accurate landings. We hypothesized that the dung beetles' need to fly considerable distances towards sporadic sources of scent, and their lack of a need to hover (or for precise landing), favour stiffer wings, whereas flower chafers spend more time hovering, landing and flying at low speed from flower to flower, favouring more flexible wings. Although this notion finds some support in our work, it warrants further investigation of the relationship between insect wing elasticity and the aerodynamic performance of the wings at low and fast flight speeds within the two beetle species.

While aerodynamic performance may select for differences in local wing stiffness, other non-mutually exclusive explanations may exist for interspecies difference in wing compliance. Insects risk wing wear and collision damage when landing on swaying vegetation [53]. More flexible wings may reduce the potential for damage [54]. Dung beetles fly and land less frequently than rose chafers and use a less cluttered flight path above the vegetation and hence are less subject to collisions. In addition, wing elasticity can also serve for elastic energy storage. With appropriate kinematics that are tuned to the elastic properties of the wing, mechanical energy saving in hawkmoths, for example, can be as high as 25% [26]. In contrast, in blowflies, *ca* 20% of the stored potential elastic energy during span-wise elastic deformation cannot be recovered [55]. Regarding the varying flexibility found in the present study, an important issue, yet to be resolved, is that of whether scarab beetles with similar flapping kinematics but varying in wing stiffness differ in their inertial energy recycling. Such differences, if present, could contribute to understanding the diversification of wing shape.

The remarkable variation in insect wing morphology and the results described herein suggest that wing-vein arrangement may be nature's way of fine-tuning insect wings to optimize flight performance in light of the trade-offs between hovering and fast forward flight, and between flight performance and energy saving. Such morphological adaptations could support species radiation and play a major role in the evolutionary success of beetles in particular, and insects in general.

## Supplementary Material

Film of crash-landing scarab beetles

Reviewer comments

## Supplementary Material

A rose chafer and a dung beetle free-flying

## Supplementary Material

Supplementary material accompanying the manuscript

## Data accessibility

Data are available through the electronic supplementary material or through links referring to the data.
