# Supplementary material for: Morphological diversification has led to inter-specific variation in elastic wing deformation during flight in scarab beetles
Source: R Soc Open Sci. 2020 Apr 15;7(4):200277. doi: 10.1098/rsos.200277 (PMC7211849; doi:10.1098/rsos.200277)
Supplement: Supplementary material accompanying the manuscript [file rsos200277supp3.pdf]

- Electronic supplementary material -

Morphological diversification has led to inter-specific variation in elastic wing deformation during flight in scarab beetles

Meresman Y [a], Husak JF [b], Ben-Shlomo R [c] and Ribak G [a,d]

[a] School of Zoology, Faculty of Life Sciences, Tel Aviv University, Tel Aviv 6997801, Israel

[b] Department of Biology, University of St. Thomas, Saint Paul, Minnesota 55105

[c] Department of Biology and the Envi ronment, University of Haifa – Oranim, Tivón, Israel

[d] The Steinhardt Museum of Natural History, Israel National Center for Biodiversity Studies, Tel Aviv 6997801, Israel

# Corresponding author: gribak@tauex.tau.ac.il

Keywords: Divergent-evolution, Elastic-deformation, Functional-morphology, Flapping flight, Free-flight, Insect.

S1: High-speed films

S1.1: A high-speed film of true dung beetles, *Copris hispanus* and *Scarabaeus puncticollis*, crash-landing.

S1.2: A high-speed film of a dung beetle (*S. puncticollis*) and a rose chafer (*Protaetia cuprea*) during low-speed free-flight.

S2: Methodology

S2.1 Elaborated methodology

Experimental procedure

Dung beetles (*S. puncticollis*) were filmed during unobstructed post-take-off flight lasting 15-42 flapping cycles, in the same set-up described previously [1]. Each beetle was filmed up to eight times, depending on their cooperation. The beetles took off from a horizontal plane and the first five flapping cycles following their detachment from the substrate were discarded to avoid transients and ground-effect [2]. We selected films in which the beetles performed three symmetrical (between contralateral wings) wingbeat cycles. The symmetry enabled us to analyse only the left wing.

Filming:

Three synchronized high-speed cameras (Fastcam SA3_120 K, Photron inc.) were fitted with 50 mm lenses and set to film at 3000 fps (shutter speed 66.7 μs, resolution: 768 × 768 pixels). We placed three LED infrared projectors pointing at each camera

to provide background illumination and two 500W visible light projectors illuminated the arena from above (figure 1*a* in the main text). To calibrate the cameras spatially, either a 10 cm^3 calibration cube or an 8 cm long wand were used with the software DLTcal5 and easywand5, respectively [3,4].

Processing of digitized data

The methodology for extracting flapping kinematics and elastic wing deformations from free-flying beetles using three high-speed cameras was previously described by Mersman and Ribak [1]. Part of the description is repeated here verbatim [5] for easy access.

Flight velocity and acceleration - The instantaneous flight velocity and mean acceleration of the beetles were calculated by fitting the time-series of instantaneous positions of the prothorax (at the lower white point in figure 1*b*) with a second order polynomial function, and finding its first and second time derivatives, respectively. The mean R^2 (n = 20) for the polynomial curve fitting functions on the X, Y and Z axes were 0.989, 0.996 and 0.999, respectively.

Normalizing the flapping cycle duration - Each analysed film section comprised three flapping cycles. To control for variance in cycle duration, we divided the serial numbers of each film frame by the number of frames within a flapping cycle, to give a non-dimensional time scale of flapping cycles in the range 0–3

Extracting the instantaneous body orientation and body frame of reference - We used the three points on the thorax (Figure 1*b* in the main text) to define a Cartesian coordinate system in which the longitudinal body axis (*x*) is represented by a vector connecting the posterior thoracic point with a point half-way between the other two

points on the pronotum. The lateral body axis (y) was defined as the vector connecting the right and left points on the pronotum. The dorso-ventral body axis (z) was found from the cross-product

$$z = x \times y \qquad \text{(Eq. S2.1)}$$

The origin was defined at the wing bases and all body axes vectors were converted to a unit vector. The vector cosines of the three body axes were used as a rotation matrix to convert each wing position to the body frame of reference (x,y,z). Thus any position on the wing in the camera frame of reference $P(X,Y,Z)$ is rotated to $P'(x,y,z)$ in the body frame of reference according to:

$$P' = \begin{bmatrix} x_X & x_Y & x_Z \\ y_X & y_Y & y_Z \\ z_X & z_Y & z_Z \end{bmatrix} \begin{bmatrix} P_X \\ P_Y \\ P_Z \end{bmatrix} \qquad \text{(Eq. S2.2)}$$

The procedure is repeated for each frame, allowing extraction of the flapping kinematics of the wing in a frame of reference that moves with the rigid body.

Extracting wing flapping kinematics - The instantaneous wing tip data in the body frame of reference and in the sagittal plane *wt* (x,z) were used to find the stroke plane of the left wing from the linear least-square-error line of these positions throughout the three flapping cycles. The stroke-plane angle (SPA) is defined as the slope of the line as in Ellington [6], but in the body frame of reference.

Next, we rotated wing data by SPA about the y axis. The instantaneous flapping and deviation angles are defined as the azimuth and elevation of the wing length relative to the stroke-plane. The incidence angle (wing pitch) is defined as the rotation of the wing about its length (see figure 1*c* in the main text). The incidence angle, was measured at 0.75 of the wing length using landmark MP (figure 1*b* in the main text).

The procedure was identical to measuring the incidence angle in *P. cuprea* but in the latter the MP landmark was slightly proximal, at 0.725 the wing span.

Extracting chord-wise wing deformations - We used the leading-edge landmarks (**wb** – wing-base, **mj** – marginal-joint, and **wt** – wing-tip, figure 1 in the main text) to define a plane that represents the rigid leading edge of the wing, and calculated the deflection of the trailing edge landmarks (**RP** – radius-posterior, **MP** – media-posterior, **CuA** – cubitus-anterior, and **AA** – analis-anterior [7], figure 1 in the main text) out of (perpendicular to) this leading-edge plane. We measured elastic wing deformations by shifting all the wing landmark positions data so that the wing base (point wb in Figure 1b) was at the origin (0,0,0). We then defined two vectors connecting wb with two non-colinear points on the leading edge (mj and wt in Figure 1b). The cross-product of the two vectors defines a vector (Z_w) normal to the plane formed by the three points (yellow triangle in Figure 1b).

$$Z_w = mj \times wt \qquad \text{(Eq. S2.3)}$$

The direction of the wing chord (Y_w) is defined as the cross-product of Zw and wt

$$Y_w = Z_w \times wt \qquad \text{(Eq. S2.4)}$$

where all vectors are converted to unit vectors.

Using the vector cosines of the three wing axes (wt, Y_w, Z_w) as a rotation matrix we transformed all data points on the wing to the wing frame of reference defined by the leading edge. The transformation of a given point P from the frame of reference of the camera to the frame of reference of the leading edge (P') is

$$P' = \begin{bmatrix} wt_X & wt_Y & wt_Z \\ Yw_X & Yw_Y & Yw_Z \\ Zw_X & Zw_Y & Zw_Z \end{bmatrix} \begin{bmatrix} P_X \\ P_Y \\ P_Z \end{bmatrix} \qquad \text{(Eq. S2.5)}$$

The procedure is repeated for each film frame. By definition, the Z_w component of the transformed data points is the deflection of this point out of the plane of the rigid leading edge.

S2.2 : Assessment of measurement error in comparing wing deformation between *P. cuprea* and *S. puncticollis*.

Unlike *P. cuprea*, the prothorax of *S. puncticollis* is tilted by ~45° from the horizontal anatomical plane of the beetle; therefore, we measured this angle (figure S2.2.1) and rotated the data about the transverse axis so that the wing kinematics of the two species were always measured in the same coordinate system defined by the anatomical body axes.

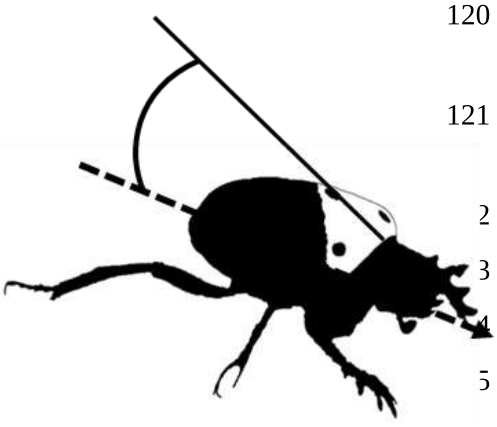

Figure S2.2.1) The tilt angle of the plane defined by the three points marked on the pronotum (continuous line) relative to the longitudinal body axis (dashed line).

The measurement of the trailing-edge landmark deflection is relative to the leading-edge plane and therefore sensitive to its definition. In beetle wings, the angle between mj and wt at wb, which defines the plane, is small, limiting the accuracy of this

definition. However, we found that the leading edge of dung beetle wings overlaps almost perfectly with those of rose chafers (Figure, S2.2.2), validating comparison of the deformation between the two species' wings.

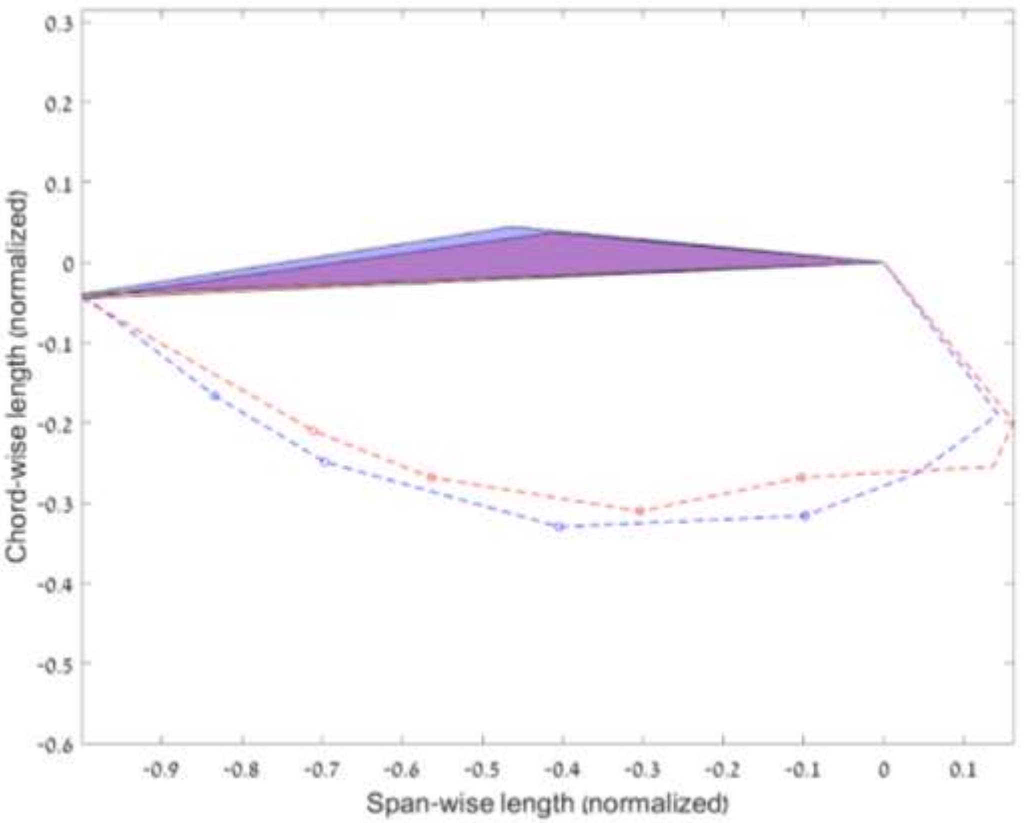

Figure S2.2.2: Alignment of the leading edge planes (shaded triangles) of *P. cuprea* (blue) and *S. puncticollis* (red). The planes are defined by the landmarks: wing-base, marginal joint and wing-tip. The trailing edge landmarks are denoted in circles. Dashed lines represent interpolation of the wing contour. The landmarks are rotated, aligned and normalized to wing length.

To elucidate measurement error arising from the wings' span-wise deflection we sampled three random films and added three landmarks between mj and wt. We found that span-wise deflection is much lower than chord-wise deflection and could account

for a maximal error of 1.63° in our measurement of the 'rigid wing' plane (figure S2.2.3).

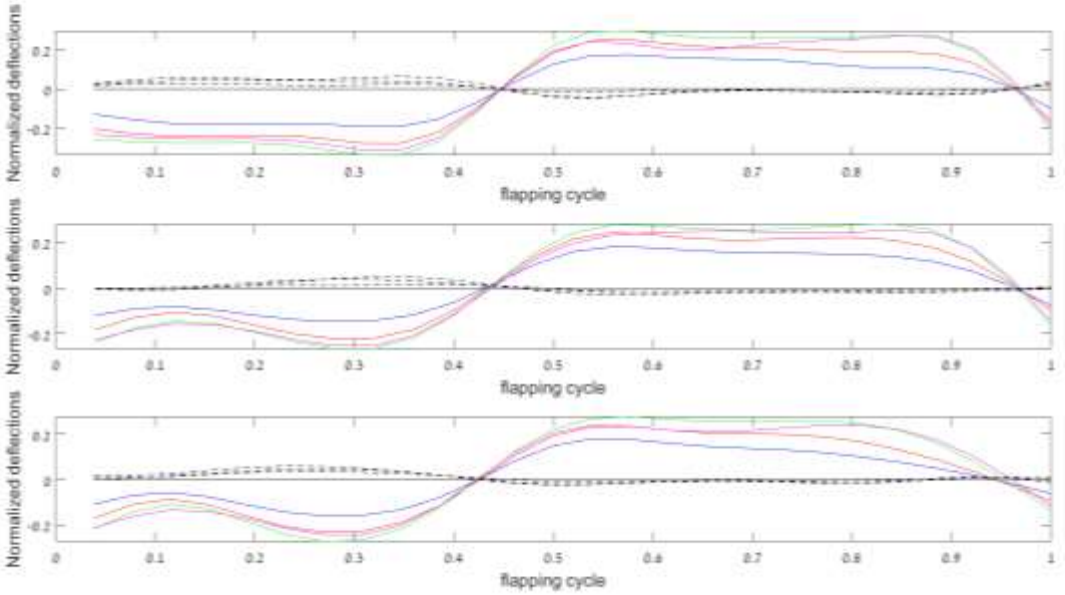

Figure S2.2.3: Chord-wise versus span-wise wing compliance during flapping in *S. puncticollis*. Normalized deflections of wing landmark out of the rigid leading-edge plane are plotted throughout a flapping cycle. The trailing-edge landmarks: RP, MP, CuA and AA are marked in blue, red, green and magenta, respectively. These chord-wise deformations are much larger than those of three additional landmarks on the distal leading-edge (between mj and wt) representing span-wise deformation of the leading edge (dashed black lines).

S2.3: Static bending measurements

S2.3.1 Experimental design

Five flower chafers and six dung beetles were randomly selected in order to measure the force needed to bend their wings. First, we anaesthetized each beetle in CO_2, and then carefully pulled the left wing from beneath the elytron and stretched it using fine forceps. Both *P. cuprea* and *S. puncticollis* fly with their elytra closed. Hence, measuring the outstretched wings, with the elytra only slightly lifted from their dorsal resting position, resembles the natural position of the elytra and flight wings during flight. After releasing the wing from the scabbard, the beetles were secured to a custom-built apparatus (figure 1 in the main text) with a small drop of quick-dry glue or hot-glue. The apparatus comprised a flat base and a vertical mount made from a microscope slide. A beetle was secured to the apparatus with its longitudinal body axis parallel to the length of the microscope slide and the wing stretched over the edge of the slide at approximately 45° from the longitudinal axis of the body. The diagonal orientation of the wing relative to the body ensured that the entire wing membrane was spread open. The wing base was secured to the edge of the slide using a mixture of baking soda and superglue, while the rest of the wing hung horizontally in the air as a cantilever beam (WB support). In the trials using the additional support (WBLE), the wings were also secured span-wise by their leading edge to another support (vertical microscope slide) using an adhesive tape, which could be peeled off at the end of the trial without damaging the wing. This measuring technique allowed us to perform the static measurements on live beetles. Preliminary trials had shown that a beetle can survive up to five days secured to the mounting device, and remains vital and feeding even a few days after detaching it from the mount. While attached, the

beetles regained movement of their limbs and head, with their spread wing remaining fixed at its base and immobile.

A force transducer (LSB200, Futek, CA, USA, accuracy ±7 mg) was fitted vertically on a horizontal arm of an XYZ micro-manipulator (Thorlabs, NJ, USA). A metallic pin, cut at the end to increase friction (tip diameter = 0.5 mm), was attached vertically to the force transducer and used to apply local pressure on the wing (figure 1 in the main text). Vertical displacement was measured visually, using a video-camera fitted with 80 mm lens, zoomed in on a small pin aligned against a grid of 1 mm squares fixed to the base (non-moving part) of the micromanipulator, giving an accuracy of 50 μm. At larger displacements (>1 mm) the pin began to slip from the wing, giving unreliable measurements.

S2.3.2: Flexural stiffness (EI) estimation

				EI (N m^2)			
Species	Point	% of span	% of chord	WB1	WBLE1	WB2	WBLE2
P. cuprea	1	15.7 (±1.8)	35.6 (±1.2)	3×10^{-7} ($\pm9\times10^{-8}$)	1×10^{-7} ($\pm4\times10^{-8}$)	3×10^{-7} ($\pm2\times10^{-7}$)	6×10^{-8} ($\pm1\times10^{-8}$)
	2	41.6 (±1.0)	35.6 (±1.2)	8×10^{-7} ($\pm1\times10^{-7}$)	2×10^{-8} ($\pm3\times10^{-9}$)	1×10^{-6} ($\pm4\times10^{-7}$)	3×10^{-8} ($\pm7\times10^{-9}$)
	3	67.6 (±0.4)	35.6 (±1.2)	6×10^{-7} ($\pm1\times10^{-7}$)	7×10^{-9} ($\pm1\times10^{-9}$)	1×10^{-6} ($\pm3\times10^{-7}$)	1×10^{-8} ($\pm3\times10^{-9}$)
	4	15.7 (±1.8)	71.2 (±2.3)	3×10^{-8} ($\pm1\times10^{-8}$)	5×10^{-8} ($\pm9\times10^{-9}$)	6×10^{-8} ($\pm4\times10^{-8}$)	7×10^{-8} ($\pm1\times10^{-8}$)
	5	41.6 (±1.0)	71.2 (±2.3)	3×10^{-7} ($\pm5\times10^{-8}$)	3×10^{-8} ($\pm3\times10^{-9}$)	4×10^{-7} ($\pm3\times10^{-7}$)	4×10^{-8} ($\pm1\times10^{-8}$)
	6	67.6 (±0.4)	71.2 (±2.3)	5×10^{-7} ($\pm1\times10^{-7}$)	1×10^{-8} ($\pm2\times10^{-9}$)	6×10^{-7} ($\pm2\times10^{-7}$)	2×10^{-8} ($\pm3\times10^{-9}$)
S. puncticollis	1	16.5 (±1.2)	42.5 (±1.0)	2×10^{-8} ($\pm1\times10^{-8}$)	3×10^{-8} ($\pm2\times10^{-8}$)	1×10^{-7} ($\pm4\times10^{-8}$)	4×10^{-8} ($\pm1\times10^{-8}$)
	2	41.7 (±0.7)	42.5 (±1.0)	2×10^{-6} ($\pm7\times10^{-7}$)	9×10^{-9} ($\pm3\times10^{-9}$)	9×10^{-7} ($\pm1\times10^{-7}$)	1×10^{-8} ($\pm3\times10^{-9}$)
	3	66.9 (±0.7)	42.5 (±1.0)	3×10^{-6} ($\pm9\times10^{-7}$)	6×10^{-9} ($\pm2\times10^{-9}$)	1×10^{-6} ($\pm2\times10^{-7}$)	1×10^{-8} ($\pm4\times10^{-9}$)
	4	16.5 (±1.2)	73.9 (±3.5)	2×10^{-8} ($\pm6\times10^{-9}$)	1×10^{-8} ($\pm5\times10^{-9}$)	5×10^{-8} ($\pm2\times10^{-8}$)	4×10^{-8} ($\pm2\times10^{-8}$)
	5	41.7 (±0.7)	81.3 (±3.5)	2×10^{-7} ($\pm8\times10^{-8}$)	1×10^{-8} ($\pm4\times10^{-9}$)	2×10^{-7} ($\pm3\times10^{-8}$)	1×10^{-8} ($\pm3\times10^{-9}$)
	6	66.9 (±0.7)	68.7 (±3.5)	6×10^{-7} ($\pm2\times10^{-7}$)	1×10^{-8} ($\pm3\times10^{-9}$)	6×10^{-7} ($\pm6\times10^{-8}$)	1×10^{-8} ($\pm3\times10^{-9}$)

S2.3.3 Statistical support

T-test statictical data for comparing force-specific deflections within and between species. WB – wing base support only, WBLE both wing base and leading edge supports. Numbers (e.g. WB2) denote whether the wing was pressed from the dorsal (1) or the ventral (2)sides.

Protaetia cuprea – Paired sample t-test

		Paired Differences							
					95% Confidence Interval of the Difference				
Point	Pair	Mean	SD	SE	Lower	Upper	t	df	P value
1	WB2 - WB1	-1.402	2.205	0.986	-4.140	1.336	-1.422	4	0.228
	WBLE1 - WBLE2	0.938	3.318	1.915	-7.303	9.179	0.490	2	0.673
	WB1 - WBLE1	-0.925	4.174	1.866	-6.107	4.258	-0.495	4	0.646
	WB2 - WBLE2	-0.583	1.519	0.877	-4.356	3.190	-0.665	2	0.574
2	WB2 - WB1	0.212	0.317	0.142	-0.181	0.605	1.496	4	0.209
	WBLE1 - WBLE2	-0.416	0.587	0.262	-1.144	0.313	-1.584	4	0.188
	WB1 - WBLE1	-0.398	0.305	0.136	-0.776	-0.019	-2.919	4	**0.043**
	WB2 - WBLE2	-0.602	0.420	0.188	-1.123	-0.080	-3.204	4	**0.033**
3	WB2 - WB1	0.047	0.036	0.018	-0.010	0.105	2.615	3	0.079
	WBLE1 - WBLE2	-0.019	0.138	0.069	-0.239	0.201	-0.273	3	0.803
	WB1 - WBLE1	-0.188	0.088	0.039	-0.297	-0.079	-4.780	4	**0.009**
	WB2 - WBLE2	-0.126	0.125	0.062	-0.325	0.072	-2.026	3	0.136
4	WB2 - WB1	0.166	0.245	0.123	-0.225	0.556	1.351	3	0.270
	WBLE1 - WBLE2	-0.017	0.073	0.037	-0.134	0.099	-0.475	3	0.667
	WB1 - WBLE1	-0.092	0.141	0.071	-0.316	0.132	-1.305	3	0.283
	WB2 - WBLE2	0.052	0.178	0.089	-0.230	0.335	0.589	3	0.597
5	WB2 - WB1	-0.033	0.059	0.029	-0.126	0.061	-1.118	3	0.345
	WBLE1 - WBLE2	-0.063	0.091	0.046	-0.209	0.082	-1.379	3	0.262
	WB1 - WBLE1	-0.005	0.013	0.007	-0.026	0.016	-0.776	3	0.494
	WB2 - WBLE2	-0.053	0.051	0.025	-0.133	0.028	-2.081	3	0.129
6	WB2 - WB1	-0.070	0.021	0.010	-0.103	-0.037	-6.805	3	**0.006**
	WBLE1 - WBLE2	-0.030	0.021	0.010	-0.057	-0.004	-3.174	4	**0.034**
	WB1 - WBLE1	0.079	0.025	0.011	0.048	0.110	7.010	4	**0.002**
	WB2 - WBLE2	-0.011	0.013	0.006	-0.031	0.009	-1.776	3	0.174

Scarabaeus puncticollis – Paired sample t-test

Point	Pair	Paired Differences					t	df	P value
					95% Confidence Interval of the Difference				
		Mean	SD	SE	Lower	Upper			
1	WB2 - WB1	0.098	0.702	0.287	-0.639	0.835	0.340	5	0.747
	WBLE1 - WBLE2	-0.723	0.344	0.172	-1.269	-0.176	-4.206	3	**0.025**
	WB1 - WBLE1	0.069	0.436	0.218	-0.625	0.763	0.316	3	0.773
	WB2 - WBLE2	-0.355	0.592	0.265	-1.090	0.381	-1.340	4	0.251
2	WB2 - WB1	0.118	0.258	0.105	-0.153	0.389	1.121	5	0.313
	WBLE1 - WBLE2	-0.286	0.095	0.047	-0.437	-0.135	-6.041	3	**0.009**
	WB1 - WBLE1	-0.005	0.258	0.129	-0.415	0.405	-0.038	3	0.972
	WB2 - WBLE2	-0.160	0.140	0.063	-0.334	0.014	-2.550	4	0.063
3	WB2 - WB1	0.026	0.031	0.014	-0.012	0.064	1.898	4	0.131
	WBLE1 - WBLE2	-0.391	0.469	0.234	-1.136	0.355	-1.669	3	0.194
	WB1 - WBLE1	-0.072	0.065	0.032	-0.175	0.031	-2.220	3	0.113
	WB2 - WBLE2	-0.436	0.414	0.207	-1.095	0.223	-2.105	3	0.126
4	WB2 - WB1	0.257	0.185	0.083	0.027	0.487	3.107	4	**0.036**
	WBLE1 - WBLE2	-0.186	0.232	0.116	-0.556	0.183	-1.605	3	0.207
	WB1 - WBLE1	-0.018	0.042	0.021	-0.085	0.049	-0.856	3	0.455
	WB2 - WBLE2	0.118	0.167	0.075	-0.090	0.326	1.574	4	0.190
5	WB2 - WB1	0.006	0.034	0.017	-0.049	0.060	0.335	3	0.759
	WBLE1 - WBLE2	0.017	0.027	0.012	-0.017	0.050	1.386	4	0.238
	WB1 - WBLE1	-0.019	0.023	0.010	-0.047	0.010	-1.785	4	0.149
	WB2 - WBLE2	0.011	0.024	0.014	-0.049	0.070	.761	2	0.526
6	WB2 - WB1	0.022	0.020	0.009	-0.003	0.047	2.405	4	0.074
	WBLE1 - WBLE2	-0.027	0.064	0.029	-0.107	0.052	-.954	4	0.394
	WB1 - WBLE1	-0.034	0.032	0.014	-0.073	0.005	-2.422	4	0.073
	WB2 - WBLE2	-0.036	0.051	0.026	-0.117	0.046	-1.396	3	0.257

Independent sample t-test for comparing force specific deflection between *P. cuprea* and *S. puncticollis*

Point		t-test for Equality of Means						
							95% Confidence Interval of the Difference	
		t	df	P value	Mean Difference	Std. Error Difference	Lower	Upper
1	WB2	-1.355	4.780	0.236	-0.812	0.599	-2.375	0.750
	WB1	-2.336	4.744	0.070	-2.312	0.990	-4.898	0.274
2	WB2	-1.078	9	0.309	-0.145	0.134	-0.448	0.159
	WB1	-0.432	9	0.676	-0.051	0.117	-0.316	0.215
3	WB2	-0.264	9	0.797	-0.007	0.026	-0.065	0.052
	WB1	1.234	9	0.249	0.023	0.019	-0.019	0.065
4	WB2	-0.233	9	0.821	-0.033	0.142	-0.355	0.289
	WB1	-1.130	9	0.288	-0.098	0.087	-0.295	0.098
5	WB2	-0.927	9	0.378	-0.076	0.082	-0.262	0.110
	WB1	-3.388	9	**0.008**	-0.066	0.019	-0.109	-0.022
6	WB2	-0.146	9	0.887	-0.002	0.015	-0.035	0.031
	WB1	-10.319	9	**<0.001**	-0.080	0.008	-0.098	-0.063

S3: Taxonomic affiliation, adult feeding preference, circadian activity and elytra position during flight in the 20 beetle species.

Family	Sub-family	Species	Adult feeding preferences	Circadian activity	Elytra posture in flight
Scarabaeinae	Cetoniinae	*Aethiessa mesopotamica*	Anthophagy	Diurnal	Closed
		Oxythyrea abigail	Anthophagy	Diurnal	Closed
		Oxythyrea noemi	Anthophagy	Diurnal	Closed
		Protaetia cuprea	Anthophagy	Diurnal	Closed
		Tropinota squalida	Anthophagy	Diurnal	Closed
	Dynastinae	*Oryctes agamemnon*	Phytophagy	Diurnal	Open
		Pentodon algerinium	Phytophagy	Diurnal	Open
		Phyllognathus excavatus	Phytophagy	Diurnal	Open
	Melolonthinae	*Anoxia laevimacula*	Phytophagy	Nocturnal	Open
		Anoxia orientalis	Phytophagy	Nocturnal	Open
		Aplidia chifensis	Phytophagy	Nocturnal	Open
		Maladera insanabilis	Phytophagy	Nocturnal	Open
	Rutelinae	*Adoretus ludmilae*	Phytophagy	Nocturnal	Open
		Blitopertha nigripennis	Phytophagy	Diurnal	Open
	Scarabaeinae	*Copris hispanus*	Coprophagy	Nocturnal	Open
		Onitis alexis	Coprophagy	Nocturnal	Closed
		Onitis ezechias	Coprophagy	Diurnal	Closed
		Scarabaeus cristatus	Coprophagy	Nocturnal	Closed
		Scarabaeus puncticollis	Coprophagy	Diurnal	Closed
Glaphyridae		*Pygopleurus sp.*	Anthophagy	Diurnal	Open

S4: Phylogenetic analysis

S 4.1 –Methods

We constructed phylogenetic trees in order to obtain the relative species distances matrix for incorporation into the GM analyses. We isolated DNA from all the tested species using a commercial kit (DNeasy ®, Qiagen). Next, we sequenced (Sanger sequencing; Hy-labs, Rehovot, Israel) one ribosomal gene (28S) and two mitochondrial genes (16S and CO1) using the primers listed in the table below (S4.1.1). Aligned sequences are available at https://galribak.weebly.com/yonatan-meresman.html. Despite repeated sequencing and DNA cleaning attempts, the sequences obtained were relatively short (430 bp, 280 bp and 230 bp for 28S, 16S and CO1, respectively). Therefore, in order to improve the reliability of the phylogenetic trees, we downloaded DNA sequences of species from the same genera as the sampled species, from the United States National Center for Biotechnology Information (NCBI) GenBank (S4.1.2), and combined them with the sequences we had produced. We aligned the sequences from both sources and built a combined Maximum Likelihood phylogenetic tree using MEGA7 (1000 Bootstraps). The sequences from both sources interlaced (S4.2); however, the trees based on the CO1 and 28S sequences were inadequately informative due to low tree branch bootstrap scores (S4.2). We therefore focused solely on the 16S sequences. The final tree (combined with the GenBank sequences) was reconstructed in R using the "Ape" [8] and "Phangorn" [9] packages, based on the aligned sequences exported from MEGA7, giving the phylogenetic distance matrix for all the species entered. The pairwise distances of the 20 species collected in the field were then pooled and used to reconstruct a new Neighbour-Joining phylogenetic tree (100 bootstraps, see S4.3).

S 4.1.1 Primers used in the study.

	gene	F/R	Primer Name	Code	Reference	Comments
1	CO1	F	C1-J-2183 (Jerry)	5’ CAA CAT TTA TTT TGA TTT TTT GG 3’	[10]	See also: http://zsm-entomology.de/wiki/The_Beetle_D_N_A_Lab#DNA_Extraction_.2F_Purification
2		R	TL2-N-3014 (Pat)	5’ TCC AAT GCA CTA ATC TGC CAT ATT A 3’	[10]	
3	28S	F	28SFF	5’ TTA CAC ACT CCT TAG CGG AT 3’	[11]	
4		R	28SDD	5’ GGG ACC CGT CTT GAA ACA C	[11]	
5		F	28SKa	5’ ACA CGG ACC AAG GAG TCT AGC ATG 3’	[11]	
6		R	28SKb	5’ CGT CCT GCT GTC TTA AGT TAC C 3’	[11]	
7	16S		LR-N-13398 16Sar	5’ CGC CTG TTT AAC AAA AAC AT 3’	[10]	3’ end of 16S
8			16SB2	CTC CGG TTT GAA CTC AGA TCA	[11]	
9			16Sb2	TTT AAT CCA ACA TCG AGG	[11]	

S 4.1.2 Genebank accessions of the specimens included in the study.

Organism	Voucher No.	Accession		
		Cox1	16S	28S
**Glaphiridae (outgroup)**				
Pygopleurus orientalis	BMNH 834234	JN969196	JN969132	JN969224
**Cetoniinae**				
Netocia cuprea	BMNH 678460	EU084043	EF487943	EU084148
Protaetia vidua	842872	JX234278	JX234543	JX234406
Protaetia angustata	842871	JX234277	JX234542	JX234405
Protaetia sp.	isolate COL192		KF801775	
Aethiessa sp. DA-2012	842874	JX234279	JX234544	JX234407
Aethiessa floralis	842761	JX234201	JX234465	JX234336
Oxythyrea cinctella	BMNH 678461	EF487733	EF487962	EU084149
Oxythyrea funesta	678443	JX234165	JX234432	JX234304
Tropinota squalida	BM835979		HQ711594	HQ599135
Tropinota hirta	703025	JX234168	JX234433	
**Dynastinae**				
Pentodon idiota	BMNH 678452	EU084045	EF487918	EU084151
Pentodon algerinum	BMNH 694812	EF487736		JN969248
Phyllognathus dionysius	BMNH 670907	EF487737	EF487944	EU084152
Oryctes cf. boas	DA-2012 842902	JX234299	JX234563	JX234427
Oryctes sp.	MA DA-2012 842881	JX234282	JX234547	JX234410
Oryctes nasicornis	BMNH 703004		EF487922	JN969247
**Melelonthinae**				
Haplidia transversa	BMNH 678410	EU084066	EF487920	EU084190
Maladera holosericea	BMNH 670868	EF487766	EF487975	EU084216
Maladera affinis	BM 678419	DQ524575	DQ681005	DQ524804
Maladera affinis	BM 670849		DQ680899	
Maladera affinis	BM 670849	DQ524363		DQ524580
Anoxia sp.	DA-2012 842889	JX234287	JX234552	JX234415
Anoxia sardoa	842887	JX234286	JX234551	JX234414

Pachydema sp.	CMN23			KJ845128
**Rutelinae**				
Blithopertha sp.	BMNH 671502	EU084137	EF487957	EU084289
Adoretus lasiopygus	BM 677906	DQ524555	DQ680980	DQ524794
Adoretus versutus	BM 677905	DQ524554	DQ680953	DQ524793
Adoretus sp. 12	BM 677883	DQ524535	DQ680857	DQ524772
**Scarabaeinae**				
Copris lugubris		AY131860	AY131493	AY131684
Copris amyntor		AY131858	AY131491	AY131683
Copris amyntor	FO43			KJ845139
Copris amyntor				DQ430934
Copris minutus				DQ430933
Onitis alexis		AY131942	AY131599	DQ430940
Onitis caffer	BMNH 670526	EF656762	AY131600	AY131784
Scarabaeus galenus		AY131956	AY131616	AY131798
Scarabaeus deludens				DQ430921

S 4.2 - Molecular phylogenetic analysis by Maximum Likelihood method for Cox1, 16S and 28S.

Affiliation to sub-families is denoted by colours. Cetoniinae(green), Scarabaeinae (yellow), Melolonthinae (violet), Rutelinae (cyan), Dynastinae (red) and Glaphiridae (black). Higher resolution figure can be downloaded at: https://galribak.weebly.com/scarab-phylogeny.html

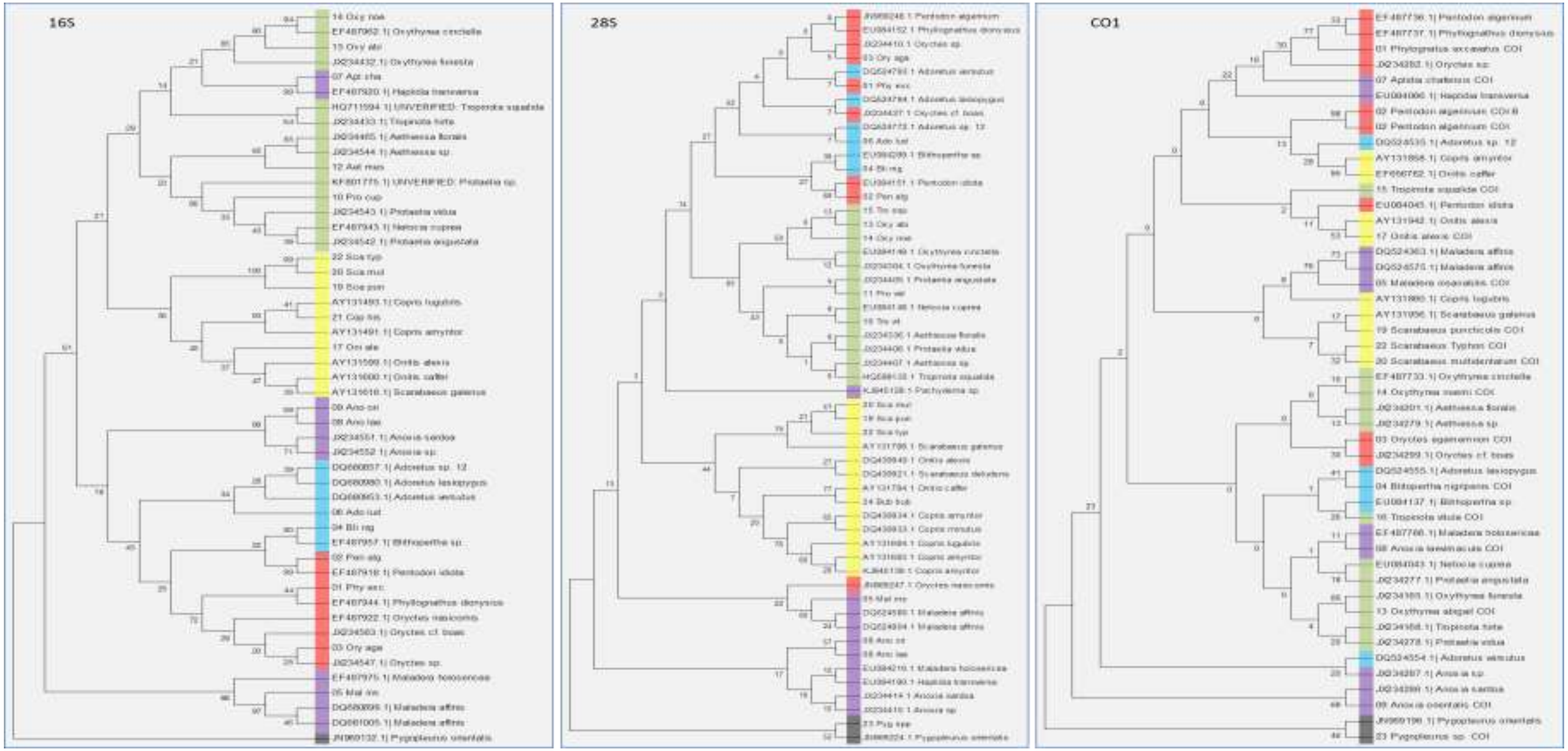

The tree branches comprise both our sequenced data and data downloaded from the NCBI Gene-Bank. The evolutionary history was inferred using the Maximum Likelihood method based on the Tamura-Nei model [12]. The bootstrap consensus tree inferred from 1,000 replicates [13] is taken to represent the evolutionary history of the analysed taxa [13]. Branches corresponding to partitions reproduced in less than 50% of bootstrap replicates are collapsed. The percentage of replicate trees in which the associated taxa clustered together in the bootstrap test (1,000 replicates) is shown next to the branches [13]. Initial tree(s) for the heuristic search were obtained automatically by applying Neighbor-Join and BioNJ algorithms to a matrix of pairwise distances estimated using the Maximum Composite Likelihood (MCL) approach, and then selecting the topology with superior log likelihood value. The analysis involved 49, 53 and 47 nucleotide sequences for 16S, 28S and CO1, respectively. All positions with less than 95% site coverage were eliminated: i.e. fewer than 5% alignment gaps, missing data and ambiguous bases were allowed at any position. There were a total of 248, 217 and 74 positions in the final dataset of 16S, 28S and CO1. Evolutionary analyses were conducted in MEGA7 [14].

S 4.3 – The code used to build a phylogenetic tree from an aligned FASTA file.

```
# Load libraries:
        library (ape)
        library (phangorn)

# Read FASTA file:
        file="filename.fas"
        dna = read.dna(file = file, format = "fasta")
        dat= phyDat(dna, type = "DNA", levels = NULL)

# Create a distance matrix from all the sequences:
        dm = dist.ml(dat, "F81")
        dm_matrix=as.matrix(dm)

# If reducing the number of entries is required, then export the data to a *.csv file,
# modify it and then import it back to R, as follows:
        write.csv(dm_matrix,"dm_matrix.csv")
        temp= read.csv("dmShort.csv")
        rownames(temp)<-temp[,1]
        temp[,1]<-NULL
        dmShort=as.dist(temp)

# Build the tree using Neighbor Joining method:
        tree=NJ(dmShort)

# Prepare with modelTest
        mt <- modelTest(dat,tree = tree)
        mt[order(mt$AICc),]

# Choose best model from the table according to AICc
        bestmodel <- mt$Model[which.min(mt$AICc)]
        env = attr(mt, "env")

# and let R search the table
        fitStart = eval(get(bestmodel, env), env)
        fit = optim.pml(fitStart, rearrangement = "stochastic",
        optGamma=TRUE, optInv=TRUE, model="HKY")
        bs = bootstrap.pml(fit, bs=100, optNni=TRUE)

# Plot Bootstrap tree:
        plotBS(midpoint(fit$tree), bs, p = 50, type="p")

# Export trees:

         write.tree (phy = fit$tree,file = "Tree.tre")
         write.tree (phy = bs,file = "BS_Tree.tre")
```

S 4.4: Phylogenetic analysis and wing GM analysis compared to taxonomic division into sub-families

Neighbour-Joining rooted phylogenetic tree based on the 16S subunit of the ribosomal mtDNA. Members of the same sub-family share the same colour. Cetoniinae (green), Scarabaeinae (yellow), Melolonthinae (violet), Rutelinae (cyan), Dynastinae (red) and Glaphiridae (black).

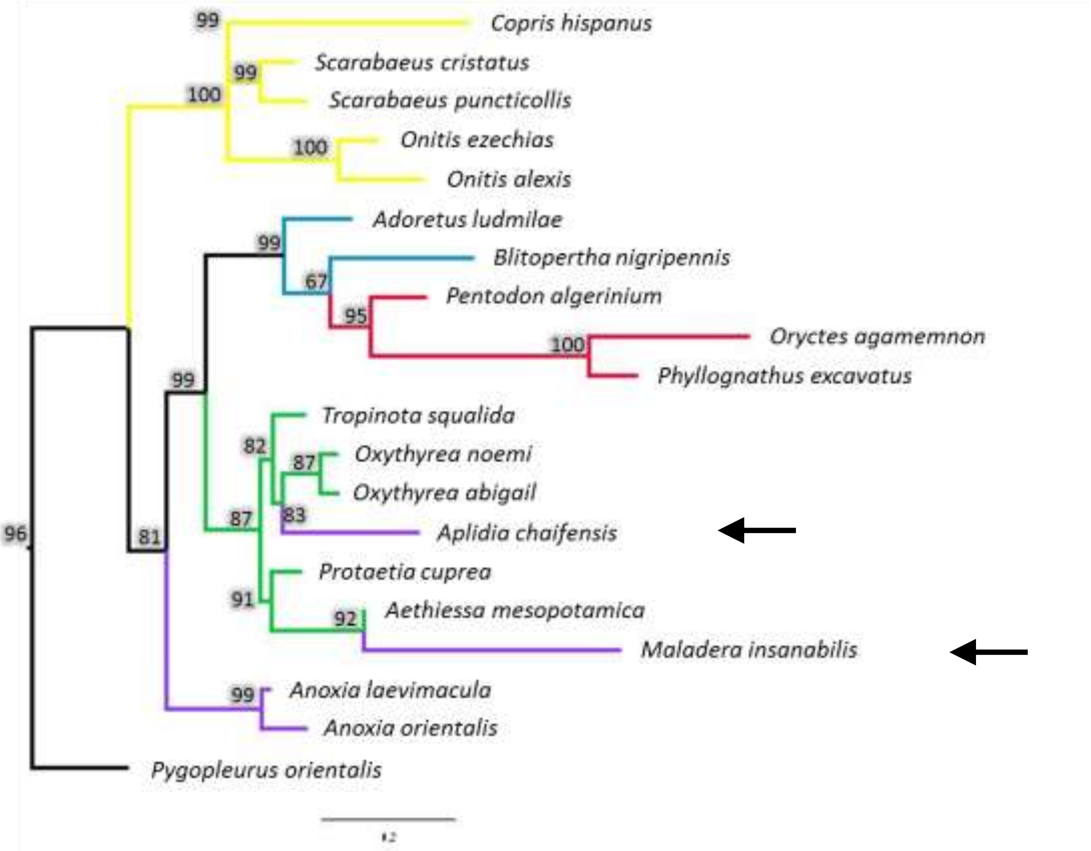

The phylogenetic classification based on the 16S sequences retrieved the Scarabaeidae family as a monophyletic clade (Bootstrap =0.96) with internal division into the monophyletic sub-families Scarabaeinae and Cetoniinae (BS = 1.0 and 0.87, respectively) and the clade of the sub-families Rutelinae and Dynastinae (hereafter: 'RD', BS = 0.99). The Dynastines are also strongly supported as monophyletic within the RD clade (BS = 0.95). In contrast, members of the sub-family Melolonthinae did not assemble into a single clade. *Anoxia laevimacula* and *A. orientalis* (tribe

melolonthini) clustered together (BS = 0.99), but *Maladera insanabilis* (tribe Sericini) and *Aplidia chaifensis* (tribe Rhizotrogini, black arrows) clustered separately with the Cetoniinae sub-family. The four members of the Melolonthinae sub-family did not cluster together in any of the three genes.

Similarly, the two sub-families (Rutelinae and Melolonthinae) were indistinguishable based on the first two PC axes (figure 6 in the main text), and also failed to demonstrate a unique wing-vein pattern that could distinguish them from other groups. This was due to the wings of *M. insanabilis* (Melolonthinae: tribe Sericini) being similar to those of *Adoretus ludmilae* (Rutelinae: Adoretini), while *Blithoperta nigripennis* (Rutelinae: Anomalini) wings were closer to those of the Melolonthinae (tribe Melolonthini) (figure 6 in the main text). Thus, both the molecular phylogenetic analysis and the wing GM analysis indicate a disagreement with the current taxonomic classification into sub-families. While we cannot rule out that the poor resolution for separating between these two sub-families was due to the small sample size, we note that our limited phylogenetic analysis is in agreement with other comprehensive phylogenetic studies that have provided a similar result [15–17].

The four Melolonthinae species in our study belong to different tribes within this sub-family. The Melolonthini and Rhizotrogini tribes include the genera *Anoxia* and *Aplidia*, respectively, which are phylogenetically closer to one another than to the *Maladera* genus from the tribe Sericini. The Sericini probably diverged from the Melolonthini and Rhizotrogini before these diverged from the Cetoniinae, Rutelinae and Dynastinae [15]; S 4.1.4). Hence, the elapsed evolutionary time since divergence of the tribes [15] supports the out-clustering of the wings of *M. insanabilis* from the rest of the Melolonthinae, as shown in our GM and phylogenetic results.

S5: Proportion of wing mass and mass moment of inertia.

Elastic wing deflection during flapping results from the aerodynamic load as well as from the inertial torque of the wing mass. The latter should be minor during mid-stroke, when we measured the deflection of the trailing edge. Nevertheless, we also compared the distribution of wing mass and mass moment of inertia along the chord-wise and span-wise axes in the two species.

Methods - Fresh wings of *S. puncticollis* and *P. cuprea* were cut into strips. Five *S. puncticollis* and 3 *P.cuprea* wings were cut parallel to wing span into 3 strips [Leading (L), central (C) and trailing (T)] and another 5 *S. puncticollis* and 5 *P.cuprea* wings were cut parallel to the chord into 4 strips (Q1 - Q4). We measured the mass of each strip and calculated the proportion of the mass (pM) for each strip from the total wing mass. The sum of masses of the strips (after cutting the wing) was on average 7% smaller compared to the initial mass of the uncut wing. We therefore corrected the masses of all strips by adding the % weight loss. We estimated the mass of each resulting wing cell by multiplying the span- and chord-wise proportions of mass (e.g., wing-base cell = $pM_{(Q1)} \times pM_{(L)}$). The relative mass of each wing cell is noted on the illustrated wings below, with warmer colours denoting a larger proportion of mass. This underlines the similarity in wing mass distribution between the species despite changes in planform shape. We also used the mass distribution to measure the proportion of the moment of inertia chord- and span-wise (Table inserts in the Figure below) according to the parallel axis theorem

$$I_W = \sum_{1}^{n} \frac{1}{12} m_n \times {w_n}^2 + m_n \times {l_n}^2$$

where m_n is the mass (in kg) of each strip, w_n is the length (in m) of each wing section and l_n is the distance (in m) between the centroids of the n-th wing section and the wing-base or leading edge in span- and chord-wise calculations, respectively. Note that although the axis for wing rotation about its length is not exactly at the leading edge, calculating the chord-wise distribution of the moment of inertia relative to the leading edge is helpful for comparing between the two wings, showing only minor differences in chord-wise moment of inertia.

Distribution of normalized mass (numbers and colour on the wings) and moment of inertia (tables) within the wings of *S. puncticollis* and *P. cuprea*: segment mass and moment of inertia are normalized by total wing mass and moment of inertia, respectivey.

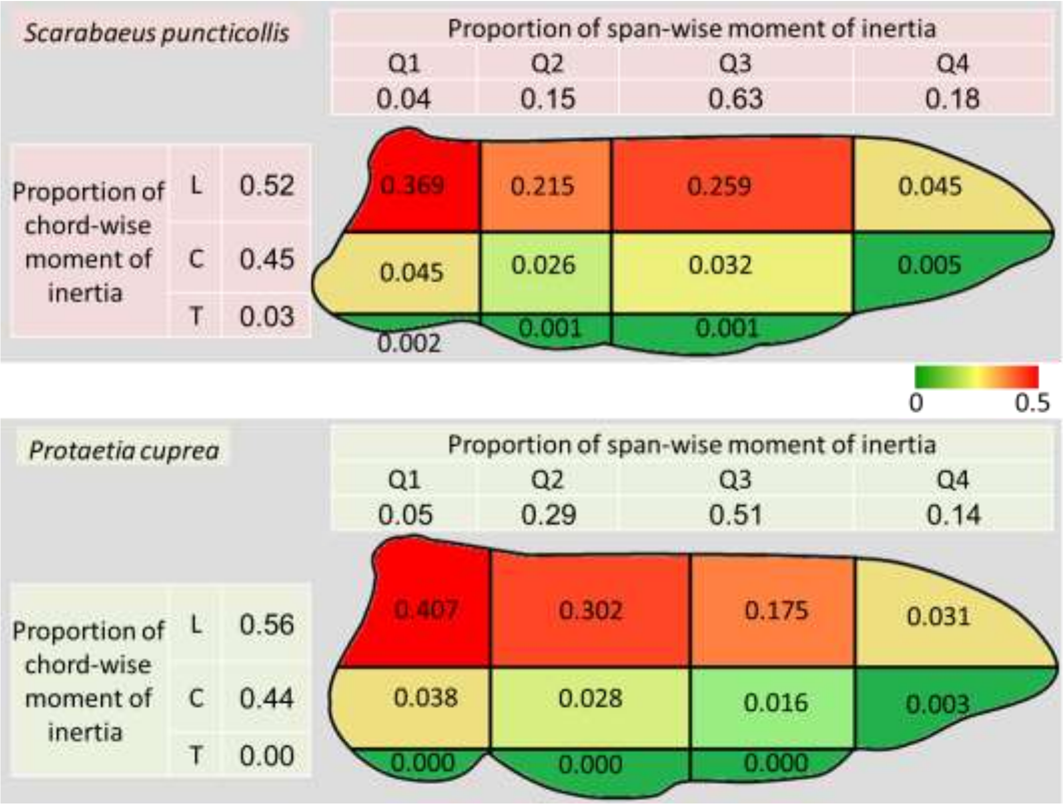

S6: Comparing the rose chafer (*Protaetia cuprea*) with the dung beetle (*Scarabaeus puncticollis*)

S6.1: mean (± SE) of studied kinematic parameters and the significance of differences between the two species.

		Species		Statistics	
Parameter	Units	*P. cuprea*	*S. puncticollis*	Test statistic	*P* value
Mass	Kg	6.9×10^{-4} (5.5×10^{-5})	5.7×10^{-4} (4.0×10^{-5})	$t_{38} = 1.856$	0.071
Wing-loading	$N \times m^{-2}$	20.26 (0.84)	20.76 (0.6)	$t_{35} = 0.493$	0.625
$\hat{r}_2$		0.529 (0.001)	0.534 (0.001)	$t_{47} = 3.793$	**<0.001**
Aspect-ratio		6.86 (0.03)	8.30 (0.06)	$t_{47} = 23.904$	**<0.001**
Stroke plane angle (β)	degrees	30 (0.7)	34 (1.5)	$t_{24.4} = 2.870$	**0.008**
Wing-beat frequency	Hz	109 (2.256)	118 (1.768)	$U = 48$	**0.001**
Flight velocity (*V*)		0.522 (0.062)	0.588 (0.024)	$t_{17.0} = 0.983$	0.340
Vertical velocity (V_z)	$m \times s^{-1}$	0.287 (0.032)	0.408 (0.033)	$U = 61$	**0.009**
Horizontal velocity (V_{xy})		0.287 (0.048)	0.365 (0.045)	$U = 105$	0.308
Vertical acceleration (a_z)	$m \times s^{-2}$	0.677 (0.314)	0.438 (0.305)	$t_{31} = 0.533$	0.598
Horizontal acceleration (a_{xy})		2.911 (0.497)	2.954 (0.348)	$t_{31} = 0.071$	0.944
Advance ratio (*J*)	$\frac{V}{2\Phi f R}$	0.056 (0.007)	0.062 (0.003)	$t_{19} = 0.749$	0.464

Φ = amplitude, *f*= frequency, *R*= wing-length

S6.2 Comparing wing flapping linematics

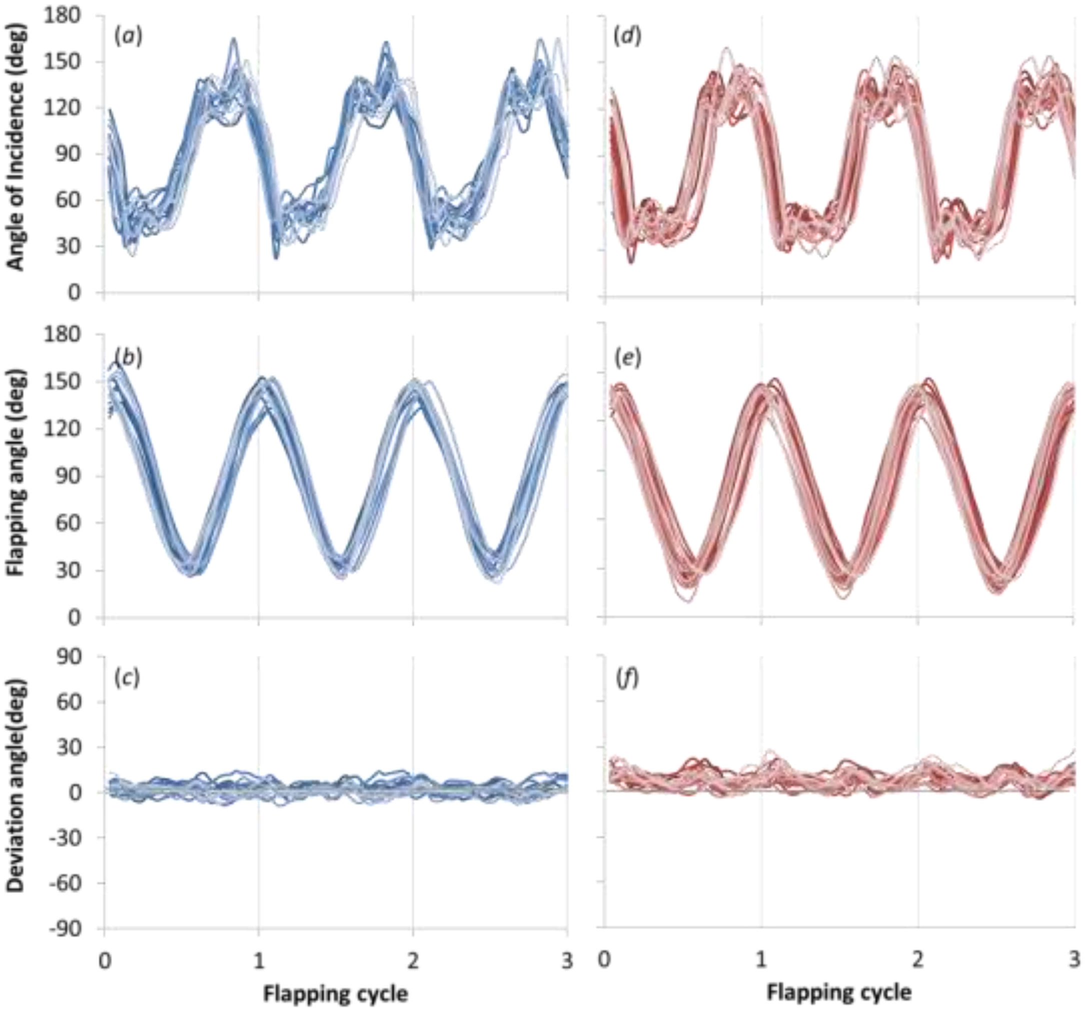

Figure S6.2: Wing flapping kinematics. Time-varying kinematics during three consecutive flapping cycles of rose chafers (a-c, blue, n = 14), and true dung beetles (d-f, red, n = 11). **a, d)** wing-pitch, **b, e)** flapping and **c, f)** deviation angles. Time is normalized by cycle duration. Each line corresponds to a single beetle. The rose chafer data are adapted from [1].

S6.3: Comparing general wing morphology

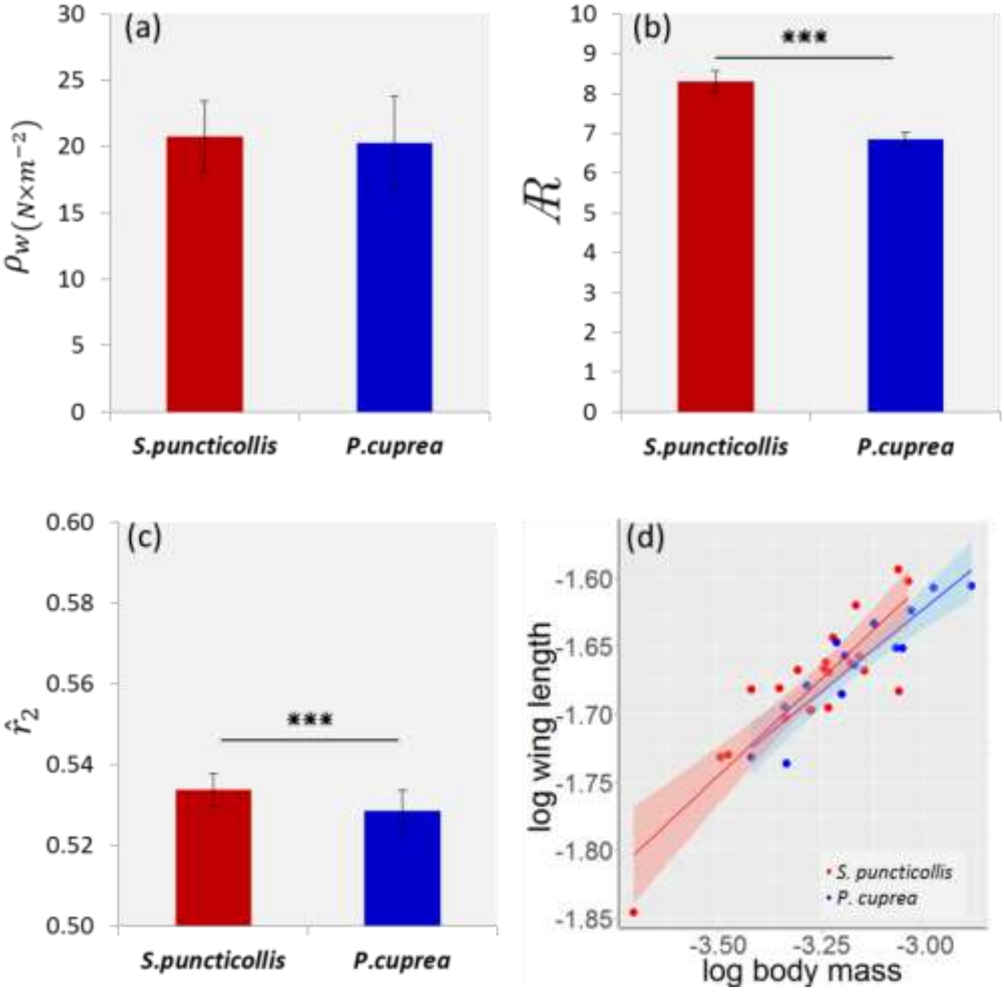

Figure S6.3: **Morphological wing properties of dung beetles (red) and rose chafers (blue).** Significant inter-species differences (p<0.001) are denoted by ***. **a)** Wing loading (t-test, t_{35} = 0.49, p = 0.625, $n_{(P.c)}$=17, $n_{(S.p)}$=20), **b)** aspect-ratio (t-test, t_{47} = 23.90, p < 0.001), **c)** non-dimensional radius of the 2nd moment of wing area (t-test, t_{47} = 3.79, p < 0.001), and **d)** Relationship between body mass and wing length. 95% confidences intervals are shaded. The species did not differ in adjusted wing length (GLM on log-transformed data with log(body mass) as a covariate; $F_{(1,31)}$ = 0.663, p = 0.422), and the interaction between wing length and body mass was not significant (F_1= 0.563, p = 0.459), suggesting similar allometric slopes. Sample sizes of *P. cuprea* and *S. puncticollis* are 29 and 20 in **a**-**c** and 14 and 20 in **d**, respectively.

S7: Supporting statistics

S7.1: Comparison between landmarks

Two-tailed comparisons of normalized deflection magnitude among the trailing edge landmarks of *S. puncticollis* during a) mid-downstroke and b) mid-upstroke. Significance levels were Bonferroni corrected to account for multiple comparisons (α = 0.0008).

a) Downstroke, paired samples two-tailed Student's t tests:

Landmark pair	Paired Differences					t	df	*P* value
	Mean	SD	SE	95% CI Lower	95% CI Upper			
RP – MP	-0.050	0.013	0.003	-0.056	-0.043	-16.323	18	<0.001
RP – CuA	-0.083	0.023	0.005	-0.095	-0.072	-15.656	18	<0.001
RP – AA	-0.070	0.024	0.006	-0.082	-0.059	-12.733	18	<0.001
MP – CuA	-0.034	0.011	0.003	-0.039	-0.028	-13.102	18	<0.001
MP – AA	-0.021	0.014	0.003	-0.027	-0.014	-6.395	18	<0.001
CuA – AA	0.013	0.011	0.002	0.008	0.018	5.249	18	<0.001

b) Upstroke, two-tailed Wilcoxon z tests:

Landmark pair	n			z	*P* value
	>	=	<		
RP : MP	0	0	19	-3.823	<0.001
RP : CuA	0	0	19	-3.823	<0.001
RP : AA	0	0	19	-3.823	<0.001
MP : CuA	0	0	19	-3.823	<0.001
MP : AA	5	0	14	-2.455	0.014
CuA – AA	18	0	1	-3.743	<0.001

S7.2: Comparison of the deflection of *P. cuprea* and *S. puncticollis* wings at specific landmarks.

	Landmark	Normalized deflection		Statistic	*p* - value
		S. puncticollis	*P. cuprea*		
Down Stroke	RP	0.128	0.090	$t_{29.4} = 5.286$	< **0.001**
	MP	0.178	0.184	$t_{27.8} = 0.587$	0.562
	CuA	0.212	0.275	$t_{31} = 4.460$	< **0.001**
	AA	0.199	0.290	$t_{31} = 6.391$	< **0.001**
Up Stroke	RP	0.128	0.074	U = 13	< **0.001**
	MP	0.187	0.158	U = 49	**0.002**
	CuA	0.240	0.252	U = 97.5	0.196
	AA	0.206	0.271	$t_{31} = 5.948$	< **0.001**

S7.3: Empirical deflection as a function of the vertical force.

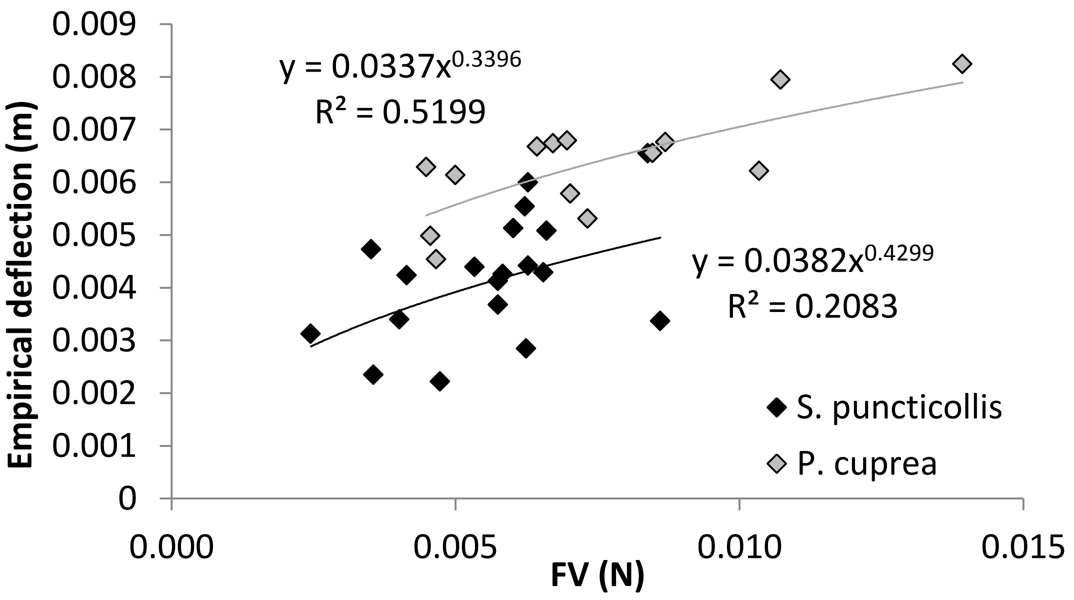

S7.4: Force distribution in the static bending experiment. The applied force (N) measured in the static bending experiment, with the wing supported in the WBLE configuration as a function of wing length. Points 1-3 and 4-6 are 2 and 4 mm away from the leading edge, respectively. Points 1 and 4 are the most proximal to the body. Blue and red circles denote *P. cuprea* and *S. puncticollis*, respectively where filled and empty circles represent data from the dorsal and ventral (WBLE1 and WBLE2) experiments, respectively.

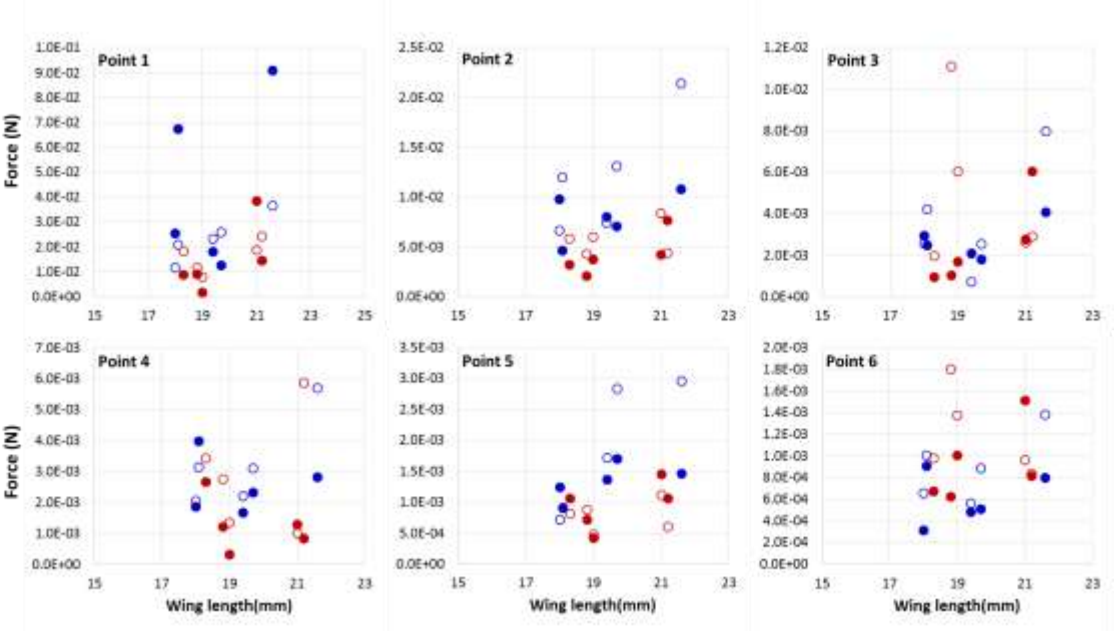

The following table summarizes the statistical results from the non-parametric Quade's Rank analysis of covariance [18]. The tests were performed using ANOVA on the residuals of the linear regression on log-transformed data.

	Quade's test for analysis of covariance	
Point	WBLE1	WBLE2
1	**$F_{1,8}$=5.406, *p* = 0.049**	**$F_{1,8}$=7.871, *p* = 0.023**
2	**$F_{1,8}$=18.03, *p* = 0.003**	**$F_{1,8}$=12.11, *p* = 0.008**
3	$F_{1,8}$=1.783, *p* = 0.218	$F_{1,8}$=0.471, *p* = 0.512
4	**$F_{1,8}$=6.477, *p* = 0.034**	$F_{1,8}$=0.121, *p* = 0.737
5	**$F_{1,8}$=5.701, *p* = 0.044**	**$F_{1,8}$=6.211, *p* = 0.037**
6	$F_{1,8}$=3.014, *p* = 0.121	$F_{1,8}$=1.179, *p* = 0.309

S7.5: Phylogenetic PCA loadings.

S7.5.1 - Loadings of the homologous landmarks (shown by numbers) calculated from the pPC1 scores.

Each landmark is plotted on its relative position on the consensus wing. Size and colour scales denote the weight of the loadings (0-2 scale).

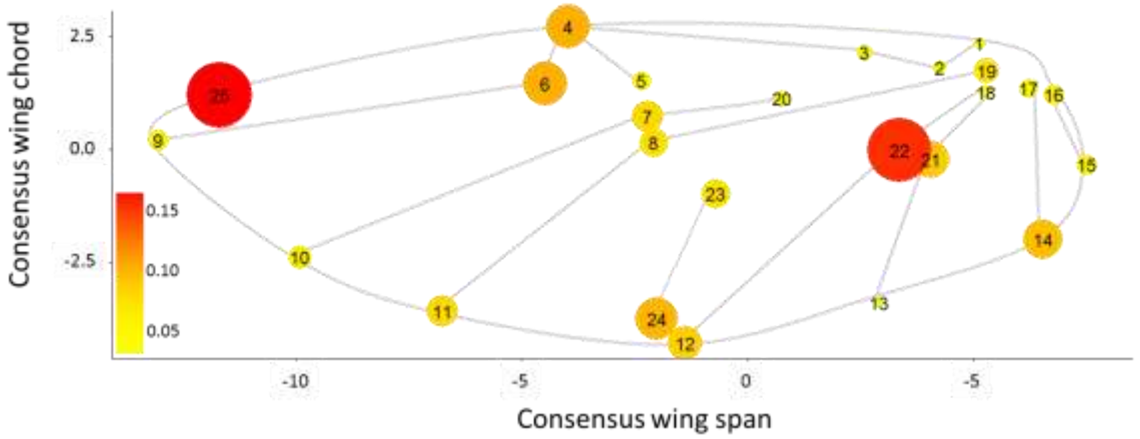

S7.5.2 – PCA loadings of the 25 landmarks (LM) for axes 1-3. The higher PCA scores of landmarks 22 and 25 in the phylogenetic PCA are highlighted.

Loadings															
Raw PCA								Phylogenetic PCA							
LM	PC1	PC2	PC3	LM	PC1	PC2	PC3		PC1	PC2	PC3	LM	PC1	PC2	PC3
1	3.44	3.51	3.47	14	4.34	4.02	12.8	1	1.47	3.85	6.23	14	5.53	3.49	4.50
2	2.71	1.58	5.12	15	2.73	4.95	5.99	2	1.85	3.30	7.34	15	2.74	2.71	7.46
3	2.58	3.92	3.67	16	1.84	2.68	2.78	3	2.19	3.06	3.74	16	2.83	6.49	6.33
4	1.43	5.86	4.25	17	2.54	3.04	1.83	4	6.12	4.92	3.62	17	2.57	7.87	4.05
5	2.69	1.93	5.71	18	2.12	2.84	2.53	5	2.58	0.94	5.75	18	1.85	5.61	4.70
6	3.13	5.08	4.63	19	2.78	2.31	3.72	6	6.09	2.67	2.65	19	3.60	5.67	5.41
7	1.11	4.12	7.85	20	1.93	5.92	2.22	7	4.44	4.81	3.52	20	2.15	5.58	5.57
8	2.58	3.59	1.58	21	5.57	5.40	2.47	8	4.05	4.55	2.66	21	5.15	2.67	2.25
9	7.54	5.59	1.58	22	7.30	8.08	2.28	9	2.88	5.71	3.84	22	8.92	1.54	1.98
10	3.01	3.08	2.51	23	2.67	4.15	2.47	10	3.19	2.90	3.75	23	4.06	3.02	2.95
11	4.41	0.53	1.86	24	8.12	5.84	3.23	11	4.41	4.04	2.25	24	5.88	4.67	0.84
12	5.51	1.86	1.78	25	15.0	3.43	4.70	12	4.85	2.47	1.28	25	9.16	3.68	2.97
13	2.91	6.70	9.00					13	1.44	3.76	4.37				

S7.6: Comparison of chordwise lengths, normalised by wing length (chord/length) at the different landmarks. The relative length is shown as mean (±SD) for *Protaetia cuprea* (n=14) and *S. puncticollis* (n=20). The data for the statistical analysis (two-tailed independent-samples t-tests) were performed on arcsine transformed proportions.

Landmark	*P. cuprea*		*S. puncticollis*	t-value	df	p-value
RP	0.212 (0.007)	<	0.261 (0.019)	10.997	26.813	**<0.001**
MP	0.306 (0.011)	ns	0.305 (0.024)	0.262	28.581	0.795
CuA	0.366 (0.019)	>	0.324 (0.026)	5.236	32	**<0.001**
AA	0.326 (0.016)	>	0.278 (0.016)	8.616	32	**<0.001**

S7.7: Flexural stiffness (*EI*) for the different landmarks based on the observed deformation during free-flight. Assuming a cantilever beam type bending and the same force applied, the flexural stiffness (*EI*) is proportional to:

$$EI \propto \frac{chord^3}{deflection}$$

Since the wings of *P. cuprea* and *S. puncticollis* vary in aspect ratio and therefore the chord length at the specific landmarks, we compared the above ratio (proportional to EI) at the homologous landmarks using the measured deflections and local chord lengths. The table below compares this ratio between the two species. Statistical invariance indicates that the *EI* is not different between the two beetle species.

The analysis shows that the ratio tends to be smaller in *P. cuprea* during the upstroke suggesting that the larger deflections observed during free-flight in the proximal wing sections of *P. cuprea* are not due solely to the longer chords but also due to reduced flexural stiffness in this region. Statistical analyses were performed on log-transformed data. Two-tailed t-tests or Mann-Whitney U test were carried out, depending on normality of the data distribution.

		P. cuprea		*S. puncticollis*	t-value	Df	p-value
Downstroke	RP	6.7×10^{-5} (2.0×10^{-5})	>	5.2×10^{-5} (1.4×10^{-5})	2.559	30	**0.016**
	MP	7.7×10^{-5} (2.3×10^{-5})	ns	6.5×10^{-5} (2.0×10^{-5})	0.013	28.802	0.990
	CuA	7.8×10^{-5} (2.5×10^{-5})	ns	8.7×10^{-5} (2.3×10^{-5})	1.251	30	0.221
	AA	5.3×10^{-5} (1.8×10^{-5})	ns	5.8×10^{-5} (1.4×10^{-5})	1.231	30	0.228
Upstroke	RP	7.0×10^{-5} (3.8×10^{-5})	ns	6.5×10^{-5} (2.0×10^{-5})	U=120		0.820
	MP	7.3×10^{-5} (3.0×10^{-5})	<	9.0×10^{-5} (2.0×10^{-5})	2.309	30	**0.028**
	CuA	6.6×10^{-5} (2.3×10^{-5})	<	9.5×10^{-5} (2.1×10^{-5})	3.861	30	**0.001**
	AA	4.9×10^{-5} (1.4×10^{-5})	<	6.2×10^{-5} (1.2×10^{-5})	3.114	30	**0.004**

S7.8: A comparison of camber (as deflection/chord length) between *P. cuprea* and *S. puncticollis*. The table summarizes two-tailed independent samples t-tests comparing the chord-specific deflection between the two species.

		$Deflection/chord\ length$					
		P. cuprea		*S. puncticollis*	t-value	Df	p-value
Downstroke	RP	0.425	ns	0.489	1.971	30	0.058
	MP	0.602	ns	0.583	-0.531	24.465	0.600
	CuA	0.752	>	0.656	-2.249	24.230	**0.034**
	AA	0.888	>	0.719	-3.468	24.458	**0.002**
Upstroke	RP	0.349	<	0.494	4.246	30	**<0.001**
	MP	0.516	<	0.617	2.758	30	**0.010**
	CuA	0.687	ns	0.750	1.847	30	0.075
	AA	0.828	>	0.749	-2.145	30	**0.040**

References for the supporting information

1. Meresman Y, Ribak G. 2017 Allometry of wing twist and camber in a flower chafer during free flight: How do wing deformations scale with body size? *R. Soc. Open Sci.* **4**, 171152. (doi:10.1098/rsos.171152)
2. Kolomenskiy D, Maeda M, Engels T, Liu H, Schneider K, Nave J-C. 2016 Aerodynamic ground effect in fruitfly sized insect takeoff. *PLoS One* **11**, e0152072. (doi:10.1371/journal.pone.0152072)
3. Theriault DH, Fuller NW, Jackson BE, Bluhm E, Evangelista D, Wu Z, Betke M, Hedrick TL. 2014 A protocol and calibration method for accurate multi-camera field videography. *J. Exp. Biol.* **217**, 1843–1848. (doi:10.1242/jeb.100529)
4. Hedrick TL. 2008 Software techniques for two- and three-dimensional kinematic measurements of biological and biomimetic systems. *Bioinspir. Biomim.* **3**, 034001. (doi:10.1088/1748-3182/3/3/034001)
5. Harriman S, Patel J. 2014 Text recycling: acceptable or misconduct? *BMC Med.* **12**, 148. (doi:10.1186/s12916-014-0148-8)
6. Ellington C. 1984 The aerodynamics of hovering insect flight. III. Kinematics. *Philos. Trans. R. Soc. Lond., B, Biol. Sci*. **305**, 41-78.
7. Haas F, Beutel RG. 2001 Wing folding and the functional morphology of the wing base in Coleoptera. *Zoology* **104**, 123–141. (doi:10.1078/0944-2006-00017)
8. Paradis E, Claude J, Strimmer K. 2004 APE: Analyses of Phylogenetics and Evolution in R language. *Bioinformatics* **20**, 289–290. (doi:10.1093/bioinformatics/btg412)
9. Schliep KP. 2011 phangorn: phylogenetic analysis in R. *Bioinformatics* **27**, 592–593. (doi:10.1093/bioinformatics/btq706)

10. Simon C, Frati F, Beckenbach A. 1994 Evolution, weighting, and phylogenetic utility of mitochondrial gene sequences and a compilation of conserved polymerase chain reaction primers. *Ann. Entomol. Soc. Am.*, **87**, 651-701
11. Monaghan MT, Inward DJG, Hunt T, Vogler AP. 2007 A molecular phylogenetic analysis of the Scarabaeinae (dung beetles). *Mol. Phylogenet. Evol.* **45**, 674–692. (doi:10.1016/j.ympev.2007.06.009)
12. Tamura K, Nei M. 1993 Estimation of the number of nucleotide substitutions in the control region of mitochondrial DNA in humans and chimpanzees. *Mol. Biol. Evol.* **10**, 512–526.
13. Felsenstein J. 1985 Confidence limits on phylogenies: An approach using the bootstrap. *Evolution* **39**, 783–791. (doi:10.1111/j.1558-5646.1985.tb00420.x)
14. Kumar S, Stecher G, Tamura K. 2016 MEGA7: Molecular Evolutionary Genetics Analysis Version 7.0 for Bigger Datasets. *Mol. Biol. Evol.* **33**, 1870–1874. (doi:10.1093/molbev/msw054)
15. Ahrens D, Schwarzer J, Vogler AP. 2014 The evolution of scarab beetles tracks the sequential rise of angiosperms and mammals. *Proc. R. Soc. B.* **281**, 20141470. (doi:10.1098/rspb.2014.1470)
16. Ahrens D. 2005 The phylogeny of Sericini and their position within the Scarabaeidae based on morphological characters (Coleoptera: Scarabaeidae). *Syst. Entomol.* **31**, 113–144. (doi:10.1111/j.1365-3113.2005.00307.x)
17. Šípek P, Fabrizi S, Eberle J, Ahrens D. 2016 A molecular phylogeny of rose chafers (Coleoptera: Scarabaeidae: Cetoniinae) reveals a complex and concerted morphological evolution related to their flight mode. *Mol. Phylogenet. Evol.* **101**, 163–175. (doi:10.1016/j.ympev.2016.05.012)

18. Quade D. 1967 Rank Analysis of Covariance. *J. Am. Stat. Assoc*. **62**, 1187–1200. (doi:10.1080/01621459.1967.10500925)
