# Supplementary material for: Morphological diversification has led to inter-specific variation in elastic wing deformation during flight in scarab beetles
Source: R Soc Open Sci. 2020 Apr 15;7(4):200277. doi: 10.1098/rsos.200277 (PMC7211849; doi:10.1098/rsos.200277)
Supplement: Reviewer comments [file rsos200277_review_history.pdf]

THE ROYAL SOCIETY
PUBLISHING

ROYAL SOCIETY
OPEN SCIENCE

Morphological diversification has led to inter-specific variation in elastic wing deformation during flight in scarab beetles

Y. Meresman, J. F. Husak, R. Ben-Shlomo and G. Ribak

Article citation details
R. Soc. open sci. **7**: 200277.
http://dx.doi.org/10.1098/rsos.200277

Review timeline
Original submission: 20 February 2020
Final acceptance: 9 March 2020

Note: Reports are unedited and appear as submitted by the referee. The review history appears in chronological order.

Note: This manuscript was transferred from another Royal Society journal with peer review.

Review History

Decision letter (RSOS-200277.R0)

09-Mar-2020

Dear Dr Merseman,

It is a pleasure to accept your manuscript entitled "Morphological diversification has led to inter-specific variation in elastic wing deformation during flight in scarab beetles." in its current form for publication in Royal Society Open Science.

You can expect to receive a proof of your article in the near future. Please contact the editorial office (openscience_proofs@royalsociety.org) and the production office (openscience@royalsociety.org) to let us know if you are likely to be away from e-mail contact -- if you are going to be away, please nominate a co-author (if available) to manage the proofing process, and ensure they are copied into your email to the journal.

Due to rapid publication and an extremely tight schedule, if comments are not received, your paper may experience a delay in publication.

Reports © 2020 The Reviewers; Decision Letters © 2020 The Reviewers and Editors; Responses © 2020 The Reviewers, Editors and Authors. Published by the Royal Society under the terms of the Creative Commons Attribution License http://creativecommons.org/licenses/by/4.0/, which permits unrestricted use, provided the original author and source are credited

Please see the Royal Society Publishing guidance on how you may share your accepted author manuscript at https://royalsociety.org/journals/ethics-policies/media-embargo/.

Thank you for your fine contribution. On behalf of the Editors of Royal Society Open Science, we look forward to your continued contributions to the Journal.

Kind regards,
Lianne Parkhouse
Editorial Coordinator
Royal Society Open Science
openscience@royalsociety.org

on behalf of Professor Emily Standen (Associate Editor) and Professor Kevin Padian (Subject Editor).

Follow Royal Society Publishing on Twitter: @RSocPublishing
Follow Royal Society Publishing on Facebook:
https://www.facebook.com/RoyalSocietyPublishing.FanPage/
Read Royal Society Publishing's blog: https://blogs.royalsociety.org/publishing/
